# MOABS: model based analysis of bisulfite sequencing data

**DOI:** 10.1186/gb-2014-15-2-r38

**Published:** 2014-02-24

**Authors:** Deqiang Sun, Yuanxin Xi, Benjamin Rodriguez, Hyun Jung Park, Pan Tong, Mira Meong, Margaret A Goodell, Wei Li

**Affiliations:** 1 Division of Biostatistics, Dan L. Duncan Cancer Center, Houston, TX 77030, USA; 2 Department of Molecular and Cellular Biology, Houston, TX 77030, USA; 3 Department of Pediatrics and Molecular & Human Genetics, Stem Cells and Regenerative Medicine Center, Baylor College of Medicine, Houston, TX 77030, USA

## Abstract

Bisulfite sequencing (BS-seq) is the gold standard for studying genome-wide DNA methylation. We developed MOABS to increase the speed, accuracy, statistical power and biological relevance of BS-seq data analysis. MOABS detects differential methylation with 10-fold coverage at single-CpG resolution based on a Beta-Binomial hierarchical model and is capable of processing two billion reads in 24 CPU hours. Here, using simulated and real BS-seq data, we demonstrate that MOABS outperforms other leading algorithms, such as Fisher’s exact test and BSmooth. Furthermore, MOABS analysis can be easily extended to differential 5hmC analysis using RRBS and oxBS-seq. MOABS is available at http://code.google.com/p/moabs/.

## Background

DNA methylation, an epigenetic modification affecting organization and function of the genome, plays a critical role in both normal development and disease. Until recently, the only known DNA methylation was 5-methylcytosine (5mC) at CpG dinucleotides, which is generally associated with transcriptional repression [1]. In 2009, another form of DNA methylation termed 5-hydroxymethylcytosine (5hmC) [2] was found to be involved in active demethylation [3] and gene regulation [4]. Understanding the functional role of DNA methylation requires knowledge of its distribution in the genome [5,6]. Bisulfite conversion of unmethylated Cs to Ts followed by deep sequencing (BS-Seq) has emerged as the gold standard to study genome-wide DNA methylation at single-nucleotide resolution. The most popular protocols include RRBS (Reduced Representation Bisulfite Sequencing) [7] and WGBS (Whole Genome Bisulfite Sequencing) [8] for the combination of 5mc and 5hmc, oxBS-Seq (Oxidative Bisulfite Sequencing) [9] for 5mc and TAB-Seq (Tet-assisted Bisulfite Sequencing) [10] for 5hmc, respectively. After mapping BS-seq reads to the genome, the proportion of unchanged Cs is regarded as the absolute DNA methylation level. Due to random sampling nature of BS-seq, deep sequencing (e.g. >30 fold) is usually required to reduce the measurement error. Technological advances and reduced costs have seen a significant increase in interest in BS-seq among biologists. Currently, BS-seq is widely used by small laboratories to profile cell lines and animal models [11], as well as by large consortiums such as the NIH ENCODE, Roadmap Epigenomics, The Cancer Genome Atlas (TCGA), and European BLUEPRINT to profile thousands of cell populations. Hence, it is expected that BS-seq data will continue to grow exponentially. However, despite recent progress [7,12-14], computational methods designed for issues specific to BS-seq are much less developed than those for other sequencing applications such as ChIP-Seq and RNA-seq.

The most fundamental aspects of BS-seq data analysis include read mapping and differential methylation detection. We previously developed one of the most widely used BS mapping programmed BSMAP [15]. After read mapping, the most common task is the identification of differentially methylated regions (DMRs) between samples, such as disease versus normal. Based on the biological question, DMRs can range in size from a single CpG (DMC: differentially methylated CpG) to tens of millions of bases. Although several statistical methods have been applied to DMR detection [12], among which Fisher’s exact test p-value (FETP) method [16] is the most popular, several challenges remain to be addressed. 1) Statistical Power: most previous methods are very conservative in power and require deep sequencing (e.g. 30 fold). For example, Hansen [13] recently calculated that for single CpG methylation level “*even 30× coverage yields standard error as large as 0.09*”. As a compromise, many studies assumed that neighboring CpGs have similar methylation levels, thus can be combined together within a genomic region (e.g. 1 kb) to increase the statistical power [17]. For example, BSmooth [13] performs local smoothing followed by t-test for DMR detection. While this strategy may be applicable in many cases, regional average analysis will unfortunately miss low-CpG-density DMRs that are abundant in the genome and critical for gene expression, such as TFBSs. Most TFBSs are small (i.e. < 50 bp) as implied by high-resolution ChIP-seq and ChIP-exo experiments [18] and contain few or even a single CpG(s) that are in general differentially methylated compared to surrounding ones, thus are very likely to be “overlooked” by the regional average analysis. 2) Biological Significance: previous methods use p-value for statistical significance of DMR. This p-value metric only tells whether a region is differentially methylated, but does not directly measure the magnitude of the methylation difference. A similar problem also exists in gene expression profiling, where the p-value does not directly measure the expression fold-change [19]. Since sequencing depth in BS-seq experiments can fluctuate by an order of magnitude in different loci, a very small methylation difference, although not biologically meaningful, can easily return a significant p-value if the underlying sequencing depth is deep enough. On the other hand, the nominal methylation difference, i.e. direct subtraction of two methylation ratios, suffers significantly from the random sampling error such that a large difference with low sequencing depth is not likely to be statistically meaningful. 3) Biological Variation is an essential feature of DNA methylation [20], and should be handled carefully to detect reproducible DMRs that represent the common characteristics of the sample group. However, most previous methods fail to account for biological variation between replicates, and simply pool the raw data from replicates for DMR detection. Some of the resulting DMRs may have significant differences at the mean level but might not be reproducible between replicates, and hence are “false-positives”. To our knowledge, BSmooth [13] is the first replicate-aware program that accounted for biological variation using a modified t-test.

In response to these challenges, we developed a powerful differential methylation analysis algorithm termed MOABS: Model-based Analysis of Bisulfite Sequencing data. Its source code is available as Additional file 1. MOABS uses a Beta-Binomial hierarchical model to capture both sampling and biological variations, and accordingly adjusts observed nominal methylation difference by sequencing depth and sample reproducibility. The resulting credible methylation difference (CDIF) is a single metric that combines both biological and statistical significance of differential methylation. Using both simulated and real whole-genome BS-seq data from mouse brain tissues and stem cells, we demonstrate the superior performance of MOABS over other leading methods, especially at low sequencing depth. Furthermore, one practical challenge is that BS-seq data analysis is usually computational intensive, and requires multiple steps. We therefore seamlessly integrate several major BS-seq processing procedures into MOABS, including read mapping, methylation ratio calling, identification of hypo- or hyper- methylated regions from one sample, and differential methylation from multiple samples. MOABS is implemented in C++ with highly efficient numerical algorithms, and thus is at least 10 times faster than other popular packages. For example, it takes only 24 CPU hours to detect differential methylation from 2 billion aligned reads. Together, MOABS provides a comprehensive, accurate, efficient and user-friendly solution for analyzing large-scale BS-seq data.

## Results and discussion

### Beta-Binomial hierarchical model for both sampling and biological variations

For a single CpG locus in the *j*-th biological replicate of condition *i*, we denote the number of total reads, the number of methylated reads and methylation ratio as *n*_*ij*_, *k*_*ij*_ and *p*_*ij*_, respectively. For a typical two group comparison, *i* = 1,2 and *j* = 1, 2, …, *N*, where *N* is the number of replicates in each condition. The *n*_*ij*_ and *k*_*ij*_ are observations from experiments, while the *p*_*ij*_ is unknown with *k*_*ij*_/*n*_*ij*_ as its nominal estimation. Given *p*_*ij*_and *n*_*ij*_, the number of methylated reads *k*_*ij*_ is characterized by the sampling variation from sequencing and can be modeled by a Binomial distribution: *k*_*ij*_ ~ *Binomial*(*n*_*ij*_, *p*_*ij*_). The posterior distribution of the methylation ratio *p*_*ij*_ will then follow a Beta distribution *Beta*(*α*_*ij*_, *β*_*ij*_)  and can be estimated using an Empirical Bayes approach. The prior distribution will be estimated from the whole genome, in which most CpGs are either fully methylated or fully un-methylated, resulting in a bimodal distribution. The Empirical Bayes approach will automatically incorporate such bimodal information in the methylation ratio estimation and hence increases the power of our analysis.

**Figure 1:**
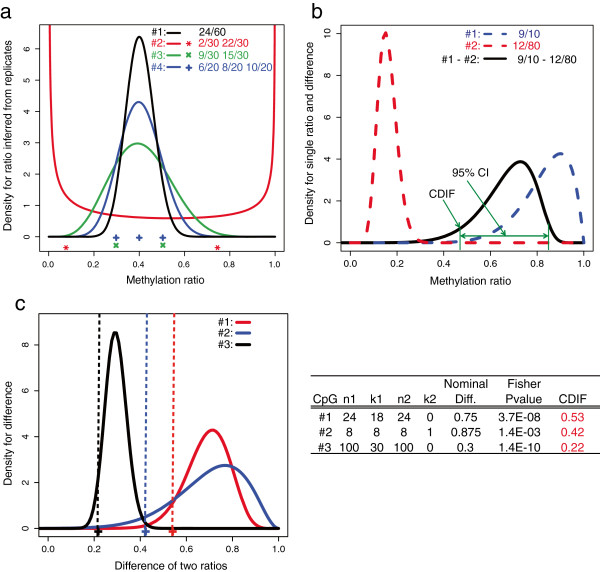
**Overview of the MOABS algorithm. (a)** Posterior distribution of methylation ratio inferred from biological replicates. Each curve represents the inferred methylation ratio Beta distribution of a CpG. The symbols at the bottom indicate the observed methylation ratios of all replicates. The values on the top right corner indicate number of methylated reads over number of total reads in each replicate. **(b)** An example of Credible Methylation Difference (CDIF). Dash curves indicate inferred methylation ratio Beta distributions from low (Sample #1) or high sequencing depth (Sample #2). The black curve is the exact distribution of the methylation difference between two samples. The CDIF is shown as the lower bound of the 95% confidence interval. **(c)** Ranking of three CpG examples by CDIF, FETP p-value and nominal difference, i.e. direct subtraction of two methylation ratios. The three curves are the exact distributions of methylation differences. The corresponding CDIF values are show as vertical dash lines.

When biological replicates are available, we will refine the posterior distribution of *p*_*ij*_ with biological variation from the Bayesian perspective. Specifically, α_*i*_ and β_*i*_ will be treated as random variables with a prior distribution estimated from all the CpGs in the genome similar to the Empirical Bayes priors. We will then use maximum likelihood approach to generate the posterior distribution of p_i_. Typical posterior distributions of four CpGs are shown in Figure 1a, in which all CpGs have the same average methylation ratios and the same total number of reads. Their methylation ratios would have identical Beta distributions (black curve on CpG #1) if biological variation was not considered. Our method is able to adjust the posterior distribution of *p*_*i*_ based on observed biological variation. For example, highly variable replicates on CpG #2 results in a bimodal distribution, whereas reproducible replicates on CpG #3 leads to a normal-like distribution. Furthermore, increasing the number of reproducible replicates from 2 to 3 on CpG #4 will reduce the variation of the resulting posterior distribution. Taken together, the posterior distribution of the methylation ratio in condition *i* will be determined by its prior distribution, sequencing depth, and the degree of variation between replicates.

### Credible methylation difference (CDIF) is a single metric for both statistical and biological significance of differential methylation

We illustrate the idea of CDIF using a simple experimental design, in which only one sample (*N = 1*) is sequenced for each of the two conditions: *k*_*ij*_ ~ *Binomial*(*n*_*i*_, *p*_*i*_) and *p*_*i*_ ~ *Beta*(*α*_*i*_, *β*_*i*_), *i* = 1, 2. The Empirical Bayes priors $\alpha_{i}^{0},\beta_{i}^{0}$ of *p*_*i*_ will be estimated from all the CpGs in the genome by maximizing a marginal likelihood function using the quasi-Newton optimization method [21]. In this case, there is no biological variation, so *Beta*(*α*_*i*_, *β*_*i*_) will be only determined by the prior distribution and sequencing depth: $\alpha_{i}=k_{i}+\alpha_{i}^{0}$ and $\beta_{i}=n_{i}-k_{i}+\beta_{i}^{0}$. An example is shown in Figure 1b. Due to low sequencing depth (*k*_1_ = 9; *n*_1_ = 10), sample #1′s Beta distribution has higher variance than that of sample #2 with high sequencing depth (*k*_2_ = 12; *n*_2_ = 80). The methylation ratio difference between two samples is denoted as *t* = *p*_*1*_ - *p*_*2*_. One immediate question is how to estimate the confidence interval *CI*(*a,b*) of *t*. Many methods have been proposed but their merits have been subject to debate [22]. We therefore propose to use the exact numerical solution [23] to solve *CI*(*a,b*). CDIF is then defined as the distance between 0 and the 95% *CI*(*a,b*) (Figure 1b):

$$\;\mathrm{CDIF}\equiv \left\{\begin{array}{c} a,\;\mathrm{if}\;a\geq 0\\ 0,\;\mathrm{if}\;a<0<b\\ b,\;\mathrm{if}\;b\leq 0 \end{array}\right)$$

In practice, CDIF represents the conservative estimation of the true methylation difference, i.e. for 97.5% of chance the absolute value of true methylation difference is greater than or equal to that of CDIF. The CDIF value will be assigned to 0 is there is no significant difference. Constructed in this way, the CDIF value, if greater than the resolution defined as min(1/*n*_1_, 1/*n*_2_), guarantees a significant p-value from Fisher’s exact test, and at the same time represents the magnitude of methylation difference. The sequencing depth will largely influence CDIF, since bigger *n*_*i*_ will make a smaller 95% CI of the methylation difference, normally resulting in greater CDIF value.

We believe CDIF is a better metric to capture the methylation difference than statistical p-value or nominal methylation difference. Three CpG examples are shown in Figure 1c. According to p-value 1.4e-10, CpG #3 is the most significant one. However, this significant p-value, which is largely driven by the high sequencing depth, does not correctly represent the actual biological difference of 0.3, which is the smallest among three CpGs. On the other hand, if we use nominal difference, CpG #2 would be the most significant. However, its low sequencing depth makes this high nominal difference unreliable. CDIF is able to penalize the nominal difference according to its statistical significance and ranks CpG #1 as the most significant followed by CpGs #2 and #3, although CpG #1 does not have the most significant p-value or nominal difference. Taken together, CDIF reaches a well balance between statistical and biological significance and gives a more stable and biological meaningful interpretation and ranking of differential methylation.

### Functions and performance of the MOABS pipeline

**Figure 2:**
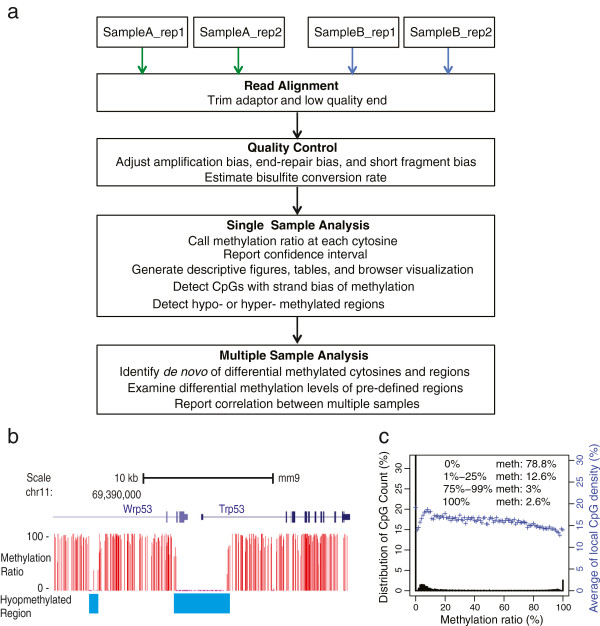
**Overview of the MOABS software pipeline. (a)** Comprehensive workflow of the MOABS pipeline. **(b)** An example of hypo-methylated region. **(c)** A descriptive figure for global methylation distribution of a mouse methylome. The Y-axis on the left is percent of CpGs and the Y-axis on the right is the average of local CpG density at each specified methylation ratio.

We have implemented MOABS as a comprehensive software pipeline (Figure 2a), including read alignment, quality control (QC), single sample analysis and multiple sample comparative analysis. 1) The read alignment model is a wrapper of popular bisulfite mapping programs, such as BSMAP [15], which allows the trimming of low quality band adaptor sequences, as well as supports parallel computing on a cluster. 2) The QC module adjusts biases in PCR amplification, end-repair, bisulfite conversion failure, and etc. [24]. In addition, it can also estimate bisulfite conversion rate based on cytosines in the non-CpG content. 3) Single sample analysis reports CpG or CpH methylation ratios with corresponding confidence intervals, detects hypo- or hyper- methylated regions (e.g. *Trp53* gene in Figure 2b) in the genome [25], and provides general statistics with descriptive figures (an example of the mouse methylome [25] is shown in Figure 2c). 3) For multiple sample comparative analysis, MOABS detects de novo DMCs, which can be further grouped into DMRs using a Hidden Markov Model. MOABS can also examine the differential methylation levels of pre-defined regions, such as promoters.

All the modules are wrapped in a single master script such that users can specify the input BS-seq reads and run all the modules one by one automatically. The MOABS pipeline is developed using C++ with highly efficient numerical algorithms, native multiple-threading and cluster support so that multiple jobs can run in parallel on different computing nodes. Several mathematical and computational optimizations have made MOABS pipeline extremely efficient. For example, it takes only one hour on 24 CPUs (IBM power7 4 Ghz) to detect differential methylation for approximately 30 million CpGs in the human genome based on 2 billion aligned reads. MOABS is significantly faster than other software. For example, a benchmark (Additional file 2: Table S1) based on public BS-seq data in mouse hematopoietic stem cell (HSC) [26] reveals that MOABS is roughly 3.3, 1.7, and 1.4 times faster than BSmooth in bisulfite mapping, methylation call and differential methylation analysis, respectively. In summary, MOABS is a comprehensive, accurate, efficient and user-friendly solution for analyzing large-scale BS-seq data.

### Simulated BS-seq data reveals the superior performance of MOABS

**Figure 3:**
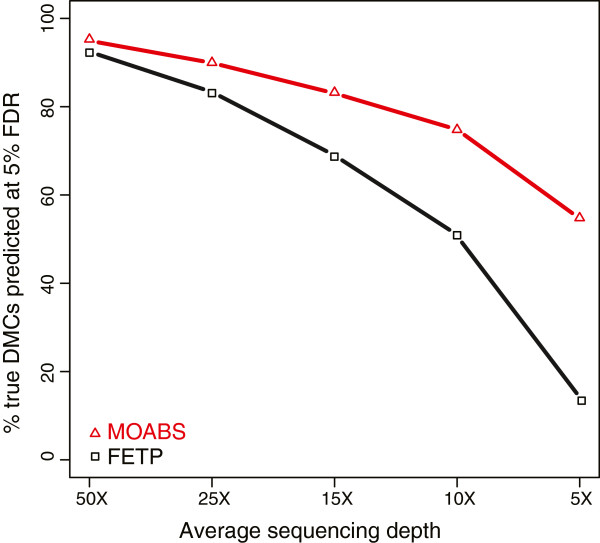
**Comparison between MOABS and FETP in detecting DMCs.** We simulated 1,000,000 CpGs in two samples with predefined true positive or true negative states. In both samples, 900,000 true negative CpGs were initially assigned the same methylation ratios. The density of the methylation ratios fits a bimodal distribution (Additional file 3: Figure S1) frequently observed in real BS-seq data. The remaining true positive 100,000 CpGs were randomly assigned at low ratios [0, 0.25] in one sample and high ratios [0.75,1] in the other sample, respectively. Each methylation ratio was then given a +/-0.05 fluctuation to simulate BS-seq errors. Sequencing depth is randomly sampled from 5-fold to 50-fold. The Y-axis shows the percentage of true DMCs predicted at 5% FDR.

To assess the performance of MOABS on differentially methylated CpGs (DMCs), we simulated 0.1 million true positive CpGs with large methylation difference and 0.9 million true negative CpGs (Additional file 3: Figure S1) from a H1 methylome [16], and then compared MOABS with FETP at 5% false discovery rate (FDR) (Figure 3). Note that this evaluation is at single CpG resolution without local smoothing, therefore BSmooth [13] cannot be used. The results indicate that MOABS clearly outperforms FETP with the most dramatic difference observed at low sequencing depth. For example, with sequencing depth at 5–10 fold, MOABS can recover 55-75% true positives while FETP only predicts 13-51% true positives. To further evaluate the performance of MOABS at different methylation levels, we re-simulated the 0.1 million true positive CpGs with different baseline methylation levels (0% -100%) and methylation differences (20% - 100%). The results (Additional file 3: Figure S2) indicate that MOABS is more accurate than FETP at any sequencing depth and at any methylation difference. Notably, the difference between the two methods is large when sequencing depth is low or when methylation difference is moderate (50% ~ 70%). In contrast, the difference between methods is small when sequencing depth is high or when the methylation difference is either very high (80% ~ 100%) or very low (~20%). Although FETP is well suited for the analysis of discrete data, it has less power for DNA methylation, which by its nature is a continuous rather than discrete random variable. The improved power of MOABS results from the modeling of DNA methylation using a Beta-Binomial hierarchical model and the Empirical Bayes approach to borrow information from all the CpGs in the genome. The testing data used for the method validation above is included in Additional file 4.

### MOABS improves the detection of allele specific DNA methylation

**Figure 4:**
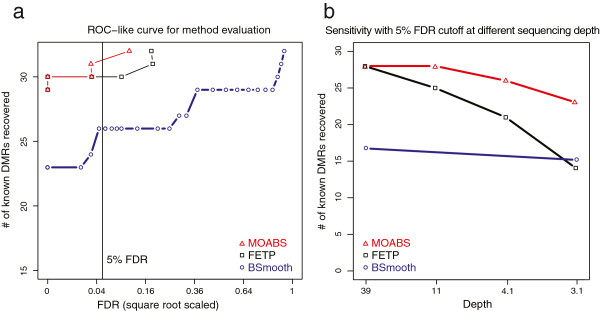
**MOABS improves the detection of allele specific DNA methylation. (a)** The y-axis shows the number of known DMRs recovered by three different methods. **(b)** Sensitivity (Y-axis) at 5% FDR with different sequencing depth (X-axis).

To assess how MOABS performs on DMRs for real BS-seq data, we compared MOABS with FETP and BSmooth [13] using allele-specific mouse methylomes [25], in which a list of well-known imprinted DMRs can serve as gold standard for method evaluation. Xie et al. [25] used FETP followed by clustering of DMCs for DMR detection. They confirmed 32 known experimentally verified imprinted DMRs (Additional file 5: Table S2) and reported 20 novel ones by pooling two biological replicates without considering sample variation. We noticed that two known DMRs (Ndn and Igf2r) are weak, exhibiting a very small methylation difference of approximately 10%. We also found that 3 novel DMRs they reported (Vwde, Casc1 and Nhlrc1) are differentially methylated in only one of the two replicates, and thus are likely to be false positives (Additional file 3: Figure S3). Since the remaining 17 novel DMRs have yet to be experimentally verified, we decided to remove them from our analysis. In our method evaluation, we used the 32 known DMRs as true positives and the remaining genome (with 17 reproducible novel DMRs removed) as true negatives. To allow for a fair comparison, we used the same method to calculate FDR for all three methods. In addition, we used the same procedure to cluster DMCs into DMRs for MOABS and FETP. The resulting ROC-like curves (Figure 4a) clearly indicate that MOABS is superior to the other two methods. MOABS successfully reports all 32 known DMRs including the two weak ones at 11% FDR, and 4 “false positive” new DMRs (Cdh20, Trappc9, Pcdhb20 and Pfdn4). Manual inspection (Additional file 3: Figure S4) confirms that these 4 “false positive” are indeed regions showing differential methylation in both replicates. Hence the 11% FDR of MOABS is significantly over estimated based on incomplete true positives. Interestingly, our FETP analysis predicts 7 new DMRs in addition to 32 known DMRs, suggesting additional filtering steps may have been performed in Xie et al. [25]. Among these 7 DMRs, one greatly overlaps with the new DMR Pcdhb20 reported by MOABS, while the other 6, including Vwde and Casc1 and Nhlc1, show poor correlation between replicates. Finally, the ROC-like curve indicates that BSmooth is less accurate than either FETP or MOABS.

The 32 known DMRs can be easily detected by both MOABS and FETP mainly because they have large methylation differences and high read depth (54-fold in DMR regions), which is consistent with our simulation study. However, deep bisulfite sequencing of the mammalian genome is still quite expensive. This reality motivated us to test to what extent these known DMRs can still be recovered at a lower sequencing depth. The same previous procedure was applied to compare all three methods. The number of recovered known DMRs at 5% FDR is plotted at each sequencing depth from random sampling (Figure 4b). We observe that the lower sequencing depth, the greater performance difference between MOABS and FETP. For example, when the depth is at 11-fold, MOABS recovers roughly 90% of known DMRs, while FETP only detects 78% of DMRs. When the depth is further lowed to 3.1-fold, MOABS can still recover roughly 70% of known DMRs, while FETP detects 44% DMRs. Interestingly, BSmooth’s performance is largely independent of sequencing depth, probably because it was designed mainly for low sequencing depth. Indeed, at a low depth of 3.1-fold, BSmooth outperforms FETP. However, at sequencing depth higher than 3.1-fold, BSmooth has a lower sensitivity than the other two methods. Collectively, we conclude that MOABS is superior in DMR detection, especially at low sequencing depth.

### MOABS reliably reveals differential methylation underlying TFBSs

**Figure 5:**
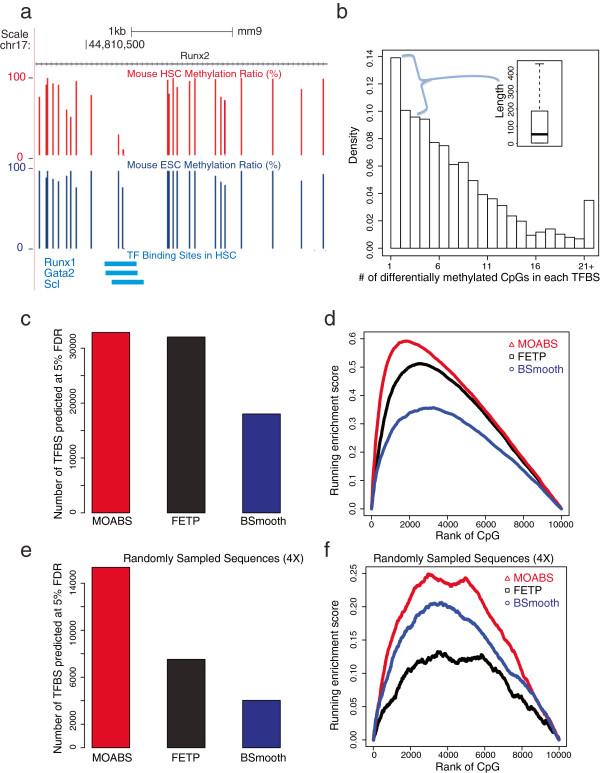
**MOABS reveals differential methylation underlying TFBSs. (a)** UCSC genome browser illustration of one TF binding site. The tracks from top to bottom are genomic positions, RefSeq Gene, HSC Methylation, ESC Methylation, and TFBS. For each CpG, an upward bar denotes the methylation ratio. **(b)** Distribution of the number of DMCs underlying TFBSs. The inserted boxplot indicates the length distribution of TFBSs with 1–3 DMCs. **(c)** Number of differentially methylated TFBSs predicted by different methods at 5% FDR. **(d)** Running enrichment scores for TFBSs. All the CpGs are ranked by each method. The score increases if the CpG is in a TFBS or decreases if not. Only 10000 CpGs are sampled to make this plot, as indicated by the x-axis. The 10000 times of random shuffle of TFBSs determined p-values of the maximum enrichment score to be 1.4E-3, 1.6E-3, and 4E-3 for MOABS, FETP and BSMOOTH respectively. **(e)** and **(f)** Same as **(c)** and **(d)** with 4X sequencing depth by random sampling. The 10000 times of random shuffle of TFBSs determined p-values of the max enrichment score to be 2.9E-2, 5.1E-2, and 9.2E-2 for MOABS, FETP and BSMOOTH respectively.

Since the previous benchmark is based on a small number of experimentally verified DMRs, we sought to further evaluate the performance of MOABS based on larger scale datasets. The link between differential methylation and TFBSs provides such a good system. TFBSs are usually hypo-methylated compared to surrounding genome background; therefore, a tissue specific TFBS is expected to be a tissue specific hypo-methylated-DMR (hypo-DMR). To test this hypothesis, we performed deep (46-fold) WGBS of the mouse hematopoietic stem cell (HSC), and compared the HSC methylome with that of a publically available mouse embryonic stem cell (ESC) [27]. The HSC methylome data is accessible at NCBI GEO Accession GSE47815. The HSC-specific hypo-DMR were then compared with approximately 58,000 *in vivo* ChIP-seq TFBSs of 10 major HSC specific TFs [28], including Erg, Fli1, Gata2, Gfi1b, Lmo2, Lyl1, Meis1, Pu.1, Runx1 and Scl. Figure 5a illustrates the hypo-methylation of a TFBS in *Runx2* gene. At the center of the TFBS co-bound by Runx1, Gata2 and Scl, there are 2 CpGs fully methylated in mouse ESC but unmethylated in HSC, while the surrounding regions are almost fully methylated in both HSC and ESC. Additional file 3: Figure S5 shows more examples of tissue specific hypo-DMR coupled with tissue specific TFBSs. Such TFBS associated hypo-methylated regions are usually very small and abundant in the genome. Using Runx1 as an example, 71% of the 4793 Runx1 TFBSs show hypo-methylation, while the remaining TFBSs are either fully methylated or have no underlying CpGs. Together, ~34% of TFBS associated hypo-methylated regions contain no more than 3 CpGs with a median length of 51 bp (Figure 5b). Furthermore, 14% of such regions even have a single CpG. For such small DMRs, single CpG level differential analysis is essential since regional averaging is very likely to overlook most of them.

We then used TFBSs to evaluate DMC detection assuming tissue-specific TF binding is associated with tissue-specific hypo-methylation. For a fair comparison, we calculated FDR for each method based on a method-specific null distribution obtained through permutation of read sample labels. At FDR of 5%, MOABS, FETP and BSmooth predicted 32,867, 32,047 and 18,021 differentially methylated TFBSs respectively (Figure 5c). We also used a method similar to Gene Set Enrichment Analysis (GSEA [29]) to test enrichment of TFBS moving down the lists of DMCs ranked by different methods. MOABS shows the highest enrichment score (Figure 5d) of TFBS. For example, with the same 4,000 most significant DMCs, MOABS recovers 1,000 TFBSs while FETP only predicts ~600 TFBSs (i.e., 40% less).

In this instance, the sequencing depth is sufficient to enable MOABS and FETP to recover very similar number of TFBSs. However, when we randomly sampled reads to a depth of 4-fold, MOABS recovered many more TFBS (15,349) than FETP (7,520) and BSmooth (4,028) (Figure 5e). Again, at this low sequencing depth, MOABS not only recovers 2–3 fold more TFBSs, but also exhibit more significant score of TFBS enrichment in the most significant DMCs. In both high and low sequencing depths, BSmooth recovers fewer TFBSs mainly because its smoothing function easily ignores small region with a few CpGs. Together, using tissue specific *in vivo* TFBSs, we demonstrate that MOABS can better recover differential methylation in small regulatory regions with a few CpGs, especially at low sequencing depth (e.g. 4-fold).

### MOABS detects differential 5hmc in ES cells using RRBS and oxBS-Seq

**Figure 6:**
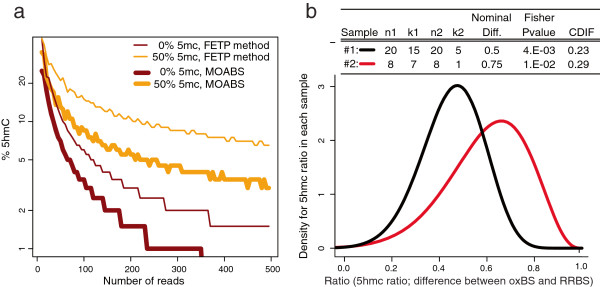
**MOABS detects differential 5hmc using RRBS and oxBS-Seq. (a)** Simulation study of 5hmc detection from oxBS-seq and RRBS. Each point on curves represents the smallest number of reads (X-axis) needed to detect a 5hmc ratio (Y-axis) at specified 5mc ratio (indicated as colors). The thin and thick curves represent FETP and MOABS, respectively. **(b)** Beta distribution of 5hmc ratio in each sample.

To demonstrate the broad utility of MOABS, we analyzed 5hmc data using RRBS and oxBS-seq [9]. RRBS measures both 5mc and 5hmc together while oxBS-Seq [9] detects 5mc directly. The 5hmc level can then be inferred by the difference between RRBS and oxBS-Seq of the same sample. The 5hmc level is often very small (e.g. at 5%) and hence its detection requires hundreds of fold coverage using FETP [9]. Our simulation study indicates that MOABS can significantly reduce the depth requirement (Figure 6a). For example, to detect 5hmc at 5% when 5mc is at 0%, MOABS requires 80-fold coverage while FETP needs ~200-fold. However, when the 5mc level is close to 50%, significantly more reads will be needed for both methods (~120-fold for MOABS and >500-fold for FETP). The differential 5hmc between two samples can be inferred by the difference of two CDIF values, each of which is the difference between RRBS and oxBS-Seq of the same sample. The similar numerical approach can then be used to infer the distribution of the difference of the difference between two Beta distributions, which are used to model BS-seq data. Figure 6b shows an example, in which 5hmc is measured by both RRBS and oxBS-Seq in two samples. FETP shows more significant p-value for 5hmc in sample #1 than in #2, whereas MOABS CDIF is bigger in sample #2 than in #1. However, the significance of FETP on sample #1 is largely driven by the high sequencing depth, thus does not correctly represent the actual biological difference. In contrast, MOABS CDIF reaches a balance between statistical and biological significance and gives a biologically meaningful differential 5hmc at CDIF value of 0.06 (0.29-0.23).

When applied to RRBS and oxBS-seq data derived from ES cell lines with different passages [9], MOABS reported 299 genes with decreased 5hmc and 125 genes with increased 5hmc (Additional file 6: Table S3) in promoters in the later passage P20, which is consistent with the mass spectrometry data [9] that shows overall reduced 5hmc in later passage. This result implies that the epigenetic stability of ES cells is impacted by prolonged *in vitro* culture. This is an important issue for both the safety and efficacy of stem cell-derived tissues in cell-replacement therapies as well as the appropriate interpretation of experimental models. Mono-allelic gene expression, including genomic imprinting, is primarily regulated through epigenetic mechanisms and thus can serve as a useful model of epigenetic stability. As expected, our analysis identified five imprinted genes with decreased 5hmc: Plagl1, Sfmbt2, Gpr1, Kcnq1 and Kcnq1ot1, as well as one imprinted gene with increased 5hmc, Pcdha4-g.

The role of 5hmc in disease remains unclear. A recent study suggests that genome-wide loss of 5hmc is an epigenetic feature of neurodegenerative Huntington’s disease [30]. The authors identified 559 genes with decreased 5hmc in the diseased mice compared to healthy controls. A considerable fraction of these disease-specific genes were uncovered in our differential 5hmc analysis in ES cells. This included 26 of 299 and 11 of 125 genes (overlapping p-value < 8e-5) with decreased and increased 5hmc, respectively. These results suggest that one potential consequence of decreased epigenetic stability over time in ES cells is the acquisition of pathological epimutations.

The observed bias toward loss of 5hmc in ES cells upon long-term culture may also suggest stem cell properties, such as pluripotency, are affected. Ficz and colleagues [31] showed that knockdown of Tet1/Tet2 in mouse ES cells down regulates epigenetic reprogramming and pluripotency-related genes such as Esrrb, Klf2, Tcl1, Zfp42, Dppa3, Ecat1 and Prdm14. Decreased expression was concomitant with both decreased 5hmC and increased 5mC at the gene promoters. In our differential 5hmc analysis in ES cells, we observed decreased 5hmc at three of these genes: Ecat1, Esrrb, and Zfp42. Together, we conclude that MOABS can be used effectively to infer differential 5hmc using RRBS and oxBS-Seq.

## Conclusions

While progress in next-generation sequencing allows increasingly affordable BS-seq experiments, the resulting data generated poses significant and unique bioinformatics challenges. The lack of efficient computational methods is the major bottleneck that prevents a broad adoption of such powerful technologies. In response to this challenge, we developed MAOBS, an accurate, comprehensive, efficient, and user-friendly pipeline for BS-seq data analysis. The MOABS analysis is novel and significant in two major aspects: 1) MOABS CDIF value provides an innovative strategy to combine statistical p-value and biological difference into a single metric, which will bring biological relevance to the interpretation of the DNA methylation data. 2) MOABS does not sacrifice resolution with low sequencing depth. By relying on the Beta-Binomial Hierarchical Model and Empirical Bayes approach, MOABS has enough power to detect single-CpG-resolution differential methylation in low-CpG-density regulatory regions, such as TFBSs, with as low as 10-fold. The low-depth BS-seq experimental design enables remarkable cost reduction per sample. In Figure 3 simulated data, we showed that MOABS achieved roughly 80% sensitivity with 5% FDR at 10-fold sequencing depth. In Figure 4b real data, we showed that as sequencing depth decreased to 11-fold by sampling, MOABS recovered roughly 90% of known DMRs. The MOABS sensitivity starts to drop dramatically when sequencing depth is further reduced. Based on the above two observations, we would recommend low-depth (e.g. 10-fold) BS-seq on more biological samples with the same limited budget, which in most scenarios will provide greater biological insights than high-depth BS-seq on fewer samples.

Copy Number Variation (CNV) is a common issue in many disease related bisulfite sequencing. The sequencing depth is normally higher or lower in high (or low) copy-number regions and this depth bias has an impact on our CDIF calculation. To correct this bias, we have included a separate script ‘redepth.pl’ in the MOABS package. Users can select their favorite CNV detection tools [32], such as CNV-Seq, Control-FREEC and VarScan, to predict the CNV region from genome sequencing or bisulfite sequencing. Nearly all these tools output a bed file of CNV regions with predicted copy number based on a p-value cutoff. The script ‘redepth.pl’ manipulates the read alignment BAM files according to the CNV prediction. If a read is located in a CNV region with a predicted copy number of X in a diploid genome, the read will have a probability of 2/X to be kept in the new BAM files. Reads in the non-CNV regions will keep unchanged. This process will result in CNV bias free BAM files for downstream analysis.

Large-scale case–control epigenome-wide association study (EWAS) is a powerful strategy to identify disease-associated epigenetic biomarkers. Currently, most studies use Illumina bisulfite arrays (e.g. 450 K) mainly due to the cost constraint. MOABS in theory can also be applied to such studies when EWAS bisulfite sequencing data are publicly available.

In summary, as DNA methylation is increasingly recognized as a key regulator of genomic function, deciphering its genome-wide distribution using BS-seq in numerous samples and conditions will continue to be a major research interest. MOABS significantly increase the speed, accuracy, statistical power and biological relevance of the BS-seq data analysis. We believe that MOABS’s superior performance will greatly facilitate the study of epigenetic regulation in numerous biological systems and disease models.

## Materials and methods

The major portions of the methods for the model are described here. In the Additional file 7, we provide more details and additional methods to make the model complete.

### Distribution for difference of two Binomial proportions

In the Additional method section (Additional file 7) we show that a methylation ratio *p* inferred from *k* methylated cytosines out of *n* total reads, follows a Beta distribution from the Bayesian perspective. The probability density function is

(1) $$f\left(p;n,k\right)=\mathrm{Be}\;\left(\alpha ,\beta \right)=\frac{p^{\alpha -1}{\left(1-p\right)}^{\beta -1}}{\int_{0}^{1}p^{\alpha -1}{\left(1-p\right)}^{\beta -1}\mathrm{dp}},$$

where α = *k* + α_0_, β = *n*-*k* + β_0_, if *Be*(α_0_, β_0_) is priori distribution for *p*. We also give formulas to numerically calculate the confidence interval for the single Binomial proportional *p* under observed (*n*, *k*).

The methylation ratio difference at a defined genomic locus from two biological samples is the difference of two Binomial proportions *p*_1_-*p*_2_. Many methods have been proposed to estimate the confidence interval *p*_1_-*p*_2_ of and their merits have been subject to decades of considerable debate [22,33-38]. No comprehensive comparison of currently available methods is available. This motivated us to turn to the direct and exact numerical calculation of confidence interval from Bayesian perspective.

Let *t* = *p*_1_-*p*_2_, where *p*_*i*_ is the proportion for the sample *i* with observation *n*_*i*_ and *k*_*i*_. Since the joint probability density of such observation is f(*p*_1_, *n*_1_, *k*_1_) f(*p*_2_, *n*_2_, *k*_2_), the PDF for *t* is

(2) $$\begin{array}{l} f\left(t\right)=\int_{0}^{1}dp_{2}f_{1}\left(p_{2}+t\right)f_{2}\left(p_{2}\right)\\ \;=\int_{0}^{1}dp_{1}f_{1}\left(p_{1}\right)f_{2}\left(p_{1}-t\right), \end{array}$$

where *f*_*i*_(*p*_*i*_) ≡ *f*(*p*_*i*_; *n*_*i*_, *k*_*i*_). Boundary conditions like the proportional area condition, minimal length condition can be applied to get unique solutions for (a, b).

### Distribution for difference of difference

Let *t* = *p*_1_ - *p*_2_, where *p*_*i*_ is the proportion for the assay *i* with observation *n*_*i*_ and k_*i*_. In the ox-BS experiments, *p*_*2*_ is the oxBS methylation ratio and *p*_1_ is the RRBS methylation ratio, and *t* is the 5hmc methylation ratio. Since the joint probability density of such observation is *f*(*p*_1_; *n*_1_; *k*_1_)*f*(*p*_2_; *n*_2_; *k*_2_), the PDF for *t* is

(3) $$\begin{array}{l} f\left(t\right)=\int_{0}^{1}dp_{2}f_{1}\left(p_{2}+t\right)f_{2}\left(p_{2}\right)\\ \;=\int_{0}^{1}dp_{1}f_{1}\left(p_{1}\right)f_{2}\left(p_{1}-t\right), \end{array}$$

where *f*_*i*_(*p*_*i*_) ≡ *f*(*p*_*i*_; *n*_*i*_, *k*_*i*_).

Let $t^{'}=p_{1}^{'}-p_{2}^{'}$, where ′ denotes the other sample. To be clear, call the two samples S and S′. In general we want to know the difference of the two 5hmc ratios, i.e., t-t′. Let *x* = t–t′, we can immediately obtain the distribution of difference of 5hmc ratio between two samples by

(4) fx=∫-11ftf't-xdt=∫-11ft'+xf't'dt',

where *f*(*t*) and *f*′(*t*′) are the distributions of 5hmc ratio for sample *S* and *S*′ respectively. After distribution of difference of 5hmc ratio between two samples is obtained, similarly confidence interval, credible difference and similarity test p-value can be calculated.

### Distribution for measurements with replicates

Here we use the exact numerical approach to calculate the distribution of *p* at observance (*m*_*i*_, *l*_*i*_) of with *m*_*i*_ as total count for replicate *i* and *l*_*i*_ as methylated count for replicate *i*. Let us start with 2 replicates. We try to fit this unknown distribution of *p* at observance (*m*_1_, *l*_1_) and (*m*_2_, *l*_2_) into a Beta distribution *f*(*p*; α,β). The parameter estimation is based on the following formula

(5) $$P\;\left(k_{i};n_{i},\alpha ,\beta \right)=\int_{0}^{1}f\left(k_{i};n_{i},p\right)f\left(p;\alpha ,\beta \right)\mathrm{dp},$$

where *P*(*k*_*i*_; *n*_*i*_, *α*, *β*) is the probability to observe (*n*_*i*_, *k*_*i*_) under the Beta distribution *f*(*p*; α, β), and *f*(*k*_*i*_; *n*_*i*_, *p*) is the Binomial distribution, i.e., the probability to observe (*n*_*i*_, *k*_*i*_) under a specific true ratio *p*. For N number of replicates, (α, β) may be estimated by maximizing the log likelihood function

(6) $$\log\;L\left(\alpha ,\beta \right)=\sum_{i=1}^{N}\log\left(C_{n_{i}}^{k_{i}}\frac{B\left(\alpha +n_{i},\;\beta +k_{i}-n_{i}\right)}{B\left(\alpha ,\beta \right)}\right),$$

where the expression inside log is the probability *P*(*k*_*i*_; *n*_*i*_, *α*, *β*) defined in equation (5) and *B* (α, β)is the Beta function.

## Abbreviations

CDIF: Credible difference; DMC: Differentially methylated cytosine; DMR: Differentially methylated region; FETP: Fisher’s exact test P-value; TFBS: Transcription factor binding site.

## Competing interests

The authors declare no competing financial interests.

## Authors’ contributions

DS and WL conceived the project, designed the algorithms, analyzed the data, and wrote the manuscript. DS developed the software package with the help of YX, TP and HJP; MM and MAG contributed the HSC methylome. All authors participated in the discussion and edited the manuscript. All authors read and approved the final manuscript.

## Supplementary Material

Additional file 1 **The source code for the software MOABS.** This version is for archive purpose only. Please download the latest version from website. Click here for file

Additional file 2: Table S1 Benchmark for performance of MOABS and BSmooth for reads alignment, methylation call, and differential methylation. Click here for file

Additional file 3 The Additional Figures S1 to S5. Click here for file

Additional file 4 The testing data used for the Credible Difference method validation. Click here for file

Additional file 5: Table S2 List of known imprinting DMRs with experimental validation references. Click here for file

Additional file 6: Table S3 List of genes with decreased 5hmc and genes with increased 5hmc. Click here for file

Additional file 7 This file is the section of additional method. Click here for file

## References

[B1] Jones PA Functions of DNA methylation: islands, start sites, gene bodies and beyond Nat Rev Genet 2012 13 484 492 10.1038/nrg3230 22641018

[B2] Tahiliani M Koh KP Shen Y Pastor WA Bandukwala H Brudno Y Agarwal S Iyer LM Liu DR Aravind L Rao A Conversion of 5-methylcytosine to 5-hydroxymethylcytosine in mammalian DNA by MLL partner TET1 Science (New York, NY) 2009 324 930 935 10.1126/science.1170116 PMC2715015 19372391

[B3] He YF Li BZ Li Z Liu P Wang Y Tang Q Ding J Jia Y Chen Z Li L Sun Y Li X Dai Q Song CX Zhang K He C Xu GL Tet-mediated formation of 5-carboxylcytosine and its excision by TDG in mammalian DNA Science 2011 333 1303 1307 10.1126/science.1210944 21817016 PMC3462231

[B4] Song CX Yi C He C Mapping recently identified nucleotide variants in the genome and transcriptome Nat Biotechnol 2012 30 1107 1116 10.1038/nbt.2398 23138310 PMC3537840

[B5] Laird PW Principles and challenges of genomewide DNA methylation analysis Nat Rev Genet 2010 11 191 203 10.1038/nrg2732 20125086

[B6] Law JA Jacobsen SE Establishing, maintaining and modifying DNA methylation patterns in plants and animals Nat Rev Genet 2010 11 204 220 10.1038/nrg2719 20142834 PMC3034103

[B7] Meissner A Mikkelsen TS Gu H Wernig M Hanna J Sivachenko A Zhang X Bernstein BE Nusbaum C Jaffe DB Gnirke A Jaenisch R Lander ES Genome-scale DNA methylation maps of pluripotent and differentiated cells Nature 2008 454 766 770 18600261 10.1038/nature07107 PMC2896277

[B8] Cokus SJ Feng S Zhang X Chen Z Merriman B Haudenschild CD Pradhan S Nelson SF Pellegrini M Jacobsen SE Shotgun bisulphite sequencing of the Arabidopsis genome reveals DNA methylation patterning Nature 2008 452 215 219 10.1038/nature06745 18278030 PMC2377394

[B9] Booth MJ Branco MR Ficz G Oxley D Krueger F Reik W Balasubramanian S Quantitative Sequencing of 5-Methylcytosine and 5-Hydroxymethylcytosine at Single-Base Resolution Science 2012 336 934 937 10.1126/science.1220671 22539555

[B10] Yu M Hon GC Szulwach KE Song CX Zhang L Kim A Li X Dai Q Shen Y Park B Min JH Jin P Ren B He C Base-resolution analysis of 5-hydroxymethylcytosine in the mammalian genome Cell 2012 149 1368 1380 10.1016/j.cell.2012.04.027 22608086 PMC3589129

[B11] Challen GA Sun D Jeong M Luo M Jelinek J Berg JS Bock C Vasanthakumar A Gu H Xi Y Liang S Lu Y Darlington GJ Meissner A Issa JP Godley LA Li W Goodell MA Dnmt3a is essential for hematopoietic stem cell differentiation Nat Genet 2012 44 23 31 22138693 10.1038/ng.1009 PMC3637952

[B12] Bock C Analysing and interpreting DNA methylation data Nat Rev Genet 2012 13 705 719 10.1038/nrg3273 22986265

[B13] Hansen KD Langmead B Irizarry RA BSmooth: from whole genome bisulfite sequencing reads to differentially methylated regions Genome Biol 2012 13 R83 10.1186/gb-2012-13-10-r83 23034175 PMC3491411

[B14] Akalin A Kormaksson M Li S Garrett-Bakelman FE Figueroa ME Melnick A Mason CE methylKit: a comprehensive R package for the analysis of genome-wide DNA methylation profiles Genome Biol 2012 13 R87 10.1186/gb-2012-13-10-r87 23034086 PMC3491415

[B15] Xi Y Li W BSMAP: whole genome bisulfite sequence MAPping program BMC Bioinforma 2009 10 232 10.1186/1471-2105-10-232 PMC2724425 19635165

[B16] Lister R Pelizzola M Dowen RH Hawkins RD Hon G Tonti-Filippini J Nery JR Lee L Ye Z Ngo Q-M Edsall L Antosiewicz-Bourget J Stewart R Ruotti V Millar AH Thomson JA Ren B Ecker JR Human DNA methylomes at base resolution show widespread epigenomic differences Nature 2009 462 315 322 10.1038/nature08514 19829295 PMC2857523

[B17] Gu H Bock C Mikkelsen TS Jäger N Smith ZD Tomazou E Gnirke A Lander ES Meissner A Genome-scale DNA methylation mapping of clinical samples at single-nucleotide resolution Nat Meth 2010 7 133 136 10.1038/nmeth.1414 PMC2860480 20062050

[B18] Rhee Ho S Pugh BF Comprehensive Genome-wide Protein-DNA Interactions Detected at Single-Nucleotide Resolution Cell 2011 147 1408 1419 10.1016/j.cell.2011.11.013 22153082 PMC3243364

[B19] Feng J Meyer CA Wang Q Liu JS Liu XS Zhang Y GFOLD: a generalized fold change for ranking differentially expressed genes from RNA-seq data Bioinformatics 2012 28 2782 2788 10.1093/bioinformatics/bts515 22923299

[B20] Feinberg AP Irizarry RA Evolution in health and medicine Sackler colloquium: Stochastic epigenetic variation as a driving force of development, evolutionary adaptation, and disease Proc Natl Acad Sci USA 2010 107 1757 1764 10.1073/pnas.0906183107 20080672 PMC2868296

[B21] Bonnans JF Gilbert JC Lemaréchal C Sagastizábal CA Numerical optimization: theoretical and practical aspects 2006 New Yor: Springer-Verlag

[B22] Kawasaki Y Comparison of exact confidence intervals for the difference between two independent binomial proportions Adv Appl Stat 2010 15 157 170

[B23] Brenner DJ Quan H Exact confidence limits for binomial proportions—Pearson and Hartley revisited J R Stat Soc. Series D (The Statistician) 1990 39 391 397

[B24] Lin X Sun D Rodriguez B Zhao Q Sun H Zhang Y Li W BSeQC: quality control of bisulfite sequencing experiments Bioinformatics 2013 29 3227 3229 10.1093/bioinformatics/btt548 24064417 PMC3842756

[B25] Xie W Barr CL Kim A Yue F Lee AY Eubanks J Dempster EL Ren B Base-resolution analyses of sequence and parent-of-origin dependent DNA methylation in the mouse genome Cell 2012 148 816 831 10.1016/j.cell.2011.12.035 22341451 PMC3343639

[B26] Jeong M Sun D Luo M Huang Y Challen GA Rodriguez B Zhang X Chavez L Wang H Hannah R Kim SB Yang L Ko M Chen R Göttgens B Lee JS Gunaratne P Godley LA Darlington GJ Rao A Li W Goodell MA Large conserved domains of low DNA methylation maintained by Dnmt3a Nat Genet 2014 46 17 23 24270360 10.1038/ng.2836 PMC3920905

[B27] Stadler MB Murr R Burger L Ivanek R Lienert F Scholer A van Nimwegen E Wirbelauer C Oakeley EJ Gaidatzis D Tiwari VK Schübeler D DNA-binding factors shape the mouse methylome at distal regulatory regions Nature 2011 480 490 495 22170606 10.1038/nature10716

[B28] Hannah R Joshi A Wilson NK Kinston S Gottgens B A compendium of genome-wide hematopoietic transcription factor maps supports the identification of gene regulatory control mechanisms Exp Hematol 2011 39 531 541 10.1016/j.exphem.2011.02.009 21338655

[B29] Mootha VK Lindgren CM Eriksson KF Subramanian A Sihag S Lehar J Puigserver P Carlsson E Ridderstrale M Laurila E Houstis N Daly MJ Patterson N Mesirov JP Golub TR Tamayo P Spiegelman B Lander ES Hirschhorn JN Altshuler D Groop LC PGC-1alpha-responsive genes involved in oxidative phosphorylation are coordinately downregulated in human diabetes Nat Genet 2003 34 267 273 10.1038/ng1180 12808457

[B30] Wang F Yang Y Lin X Wang JQ Wu YS Xie W Wang D Zhu S Liao YQ Sun Q Yang Y Guo C Han C Tang T Genome-wide loss of 5-hmC is a novel epigenetic feature of Huntington’s disease Hum Mol Genet 2013 22 3641 3653 10.1093/hmg/ddt214 23669348

[B31] Ficz G Branco MR Seisenberger S Santos F Krueger F Hore TA Marques CJ Andrews S Reik W Dynamic regulation of 5-hydroxymethylcytosine in mouse ES cells and during differentiation Nature 2011 473 398 402 10.1038/nature10008 21460836

[B32] Min Zhao QW Quan W Peilin J Zhongming Z Computational tools for copy number variation (CNV) detection using next-generation sequencing data: features and perspectives BMC Bioinforma 2013 14 S1 10.1186/1471-2105-14-S11-S1 PMC3846878 24564169

[B33] Newcombe RG Interval estimation for the difference between independent proportions: comparison of eleven methods Stat Med 1998 17 873 890 10.1002/(SICI)1097-0258(19980430)17:8<873::AID-SIM779>3.0.CO;2-I 9595617

[B34] Wilson EB Probable inference, the law of succession, and statistical inference J Am Stat Assoc 1927 22 209 212 10.1080/01621459.1927.10502953

[B35] Santner TJ Pradhan V Senchaudhuri P Mehta CR Tamhane A Small-sample comparisons of confidence intervals for the difference of two independent binomial proportions Comput Stat Data Anal 2007 51 5791 5799 10.1016/j.csda.2006.10.018

[B36] Nurminen MM Newcombe RG Score intervals for the difference of two binomial proportions http://markstat.net/en/images/stories/notes_on_score_intervals.pdf

[B37] Pradhan VB Tathagata Confidence interval of the difference of two independent binomial proportions using weighted profile likelihood Commun Stat Simul Comput 2008 37 645 659 10.1080/03610910701767721

[B38] Coe PR Tamhane AC Small sample confidence intervals for the difference,ratio and odds ratio of two success probabilities Commun Stat Simul Comput 1993 22 925 938 10.1080/03610919308813135

