# Supplementary material for: MOABS: model based analysis of bisulfite sequencing data
Source: Genome Biol. 2014 Feb 24;15(2):R38. doi: 10.1186/gb-2014-15-2-r38 (PMC4054608; doi:10.1186/gb-2014-15-2-r38)
Supplement: Additional file 3 — The Additional Figures S1 to S5. [file gb-2014-15-2-r38-S3.pdf]

Additional Figures

Figure S1. Bimodal distribution of simulated methylation ratios.
Each bar represents the percent of CpGs in simulated methylome at each specified methylation ratio..

Figure S2. DMC detection based on simulated data
The y-axis is the percent of DMCs predicted by different methods at 5% FDR. Each panel shows the results for DMC detection with different simulated methylation difference.

Figure S3. Non-reproducible DMRs predicted by FETP.
The first and second rows are for Vwde and Casc1 DMRs, respectively. The first, second and third columns show the methylation information for replicate F1i, F1r and direct combination of two replicates, respectively. Each bar represents methylation ratio of a CpG in the DMR region. Red bar shows maternal methylation and blue bar shows paternal methylation. Vwde DMR shows differential methylation in replicate F1r but not in F1i; Casc1 DMR shows differential methylation in replicate F1i but not in F1r. The reproducibility information is lost by direct combination of replicates. The Nhlrc1 DMR (chr13:47106177-47106300) is not shown due to too many CpGs.

Figure S4. Novel imprinted DMRs predicted by MOABS at level of Ndn and Igf2r.
The first, second and third rows are for Cdh20, Trappc9, and Pfdn4 DMRs respectively. The first, second and third columns show the methylation information for replicate F1i, F1r and direct combination of two replicates respectively. Each bar represents methylation ratio of a CpG in the DMR region. Red bar shows maternal methylation and blue bar shows paternal methylation. The differential methylation is reproducible in both replicates. The nominal difference is small but they are at the same level of known DMRs Ndn and Igf2r.

Figure S5. Examples of DMCs associated with TFBS.
Park2 and *Scin* show the small hypomethylated region surrounded by full methylation. The two tracks are methylation ratio in HSC and ESC with each bar denoting the methylation ratio on each CpG. The bottom track marks the positions of binding regions of TFs in mouse HSC.

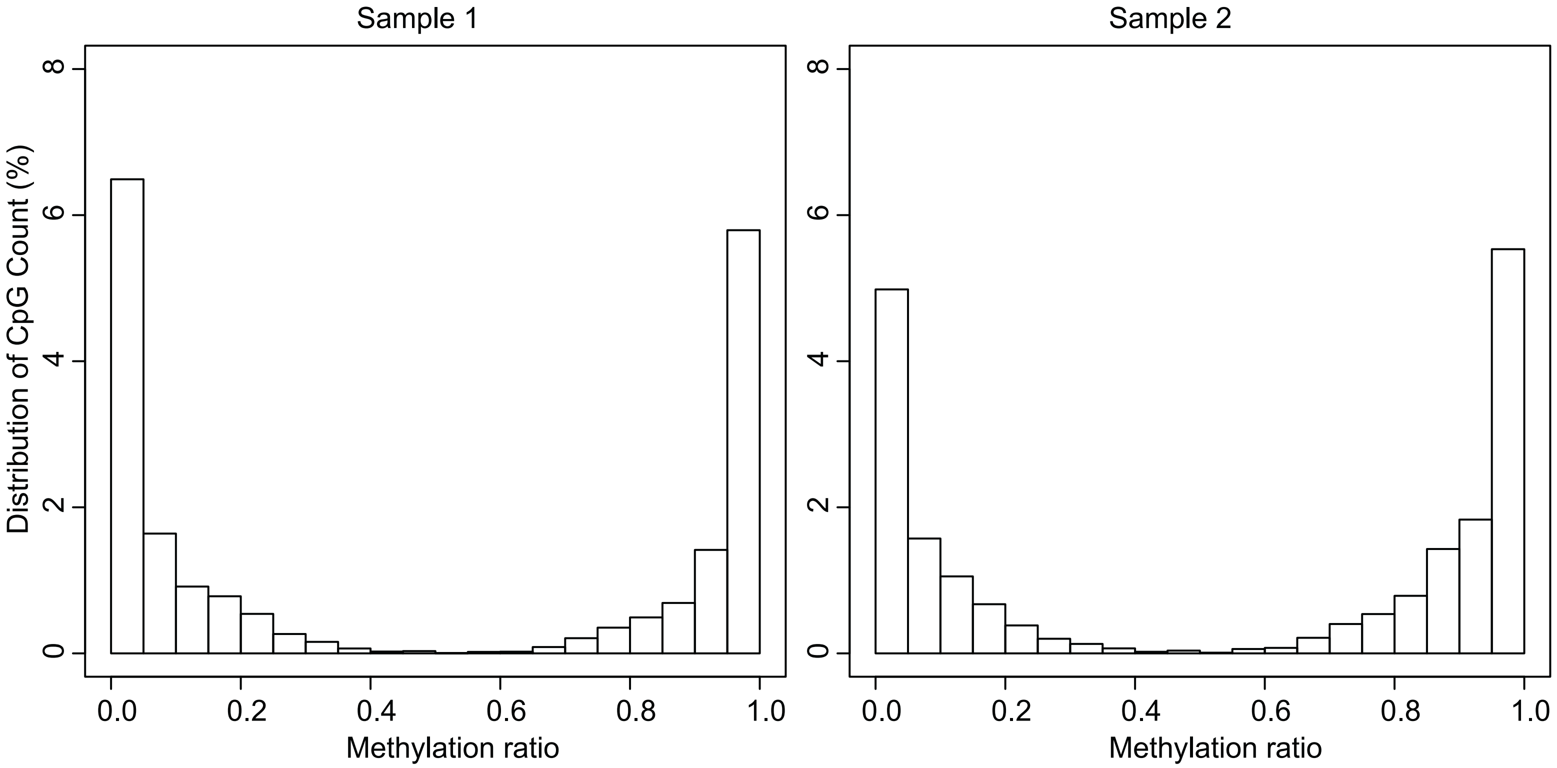

Supp Figure 1. Sun *et al.*

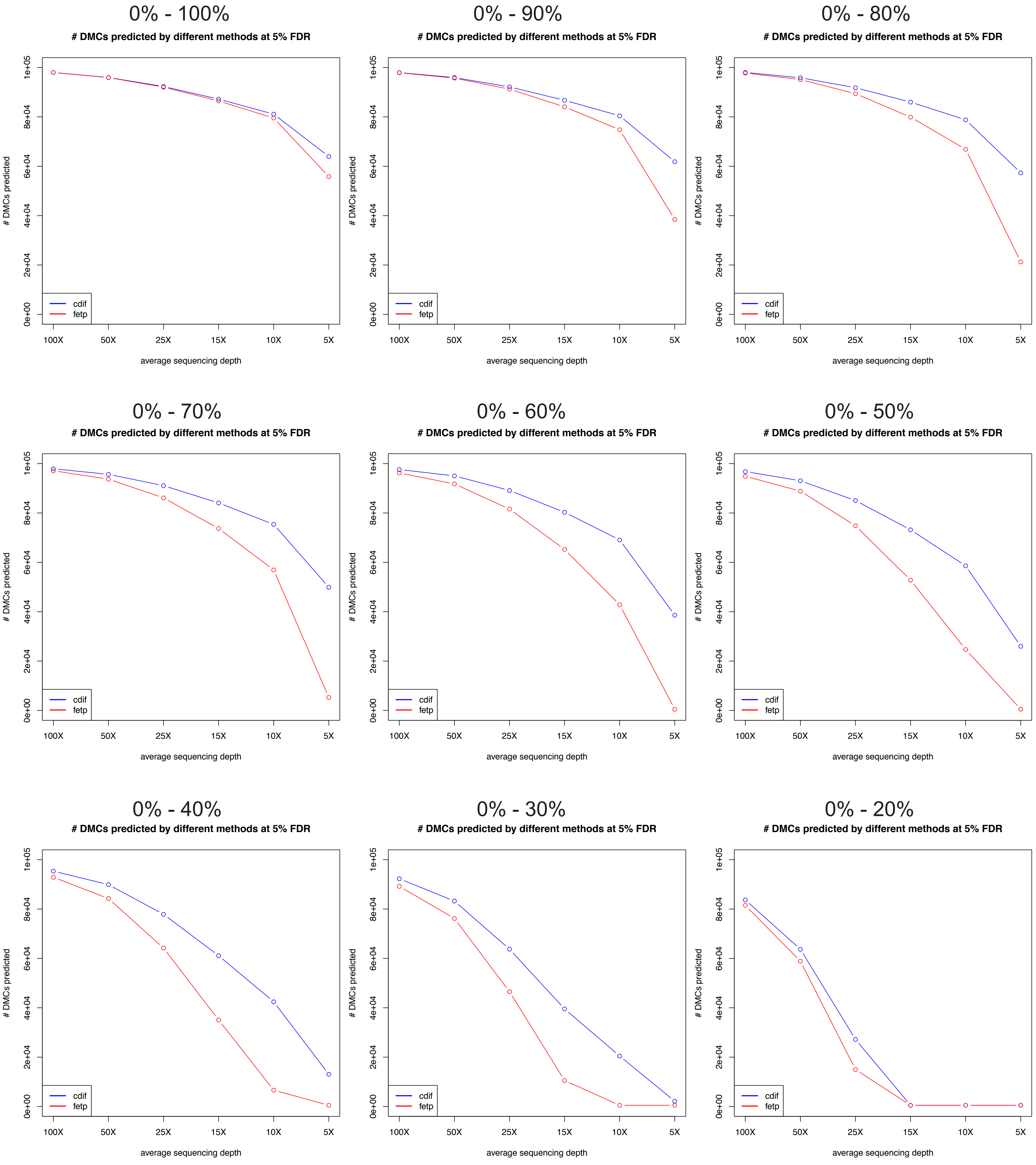

Supp Figure 2a. Sun *et al.*

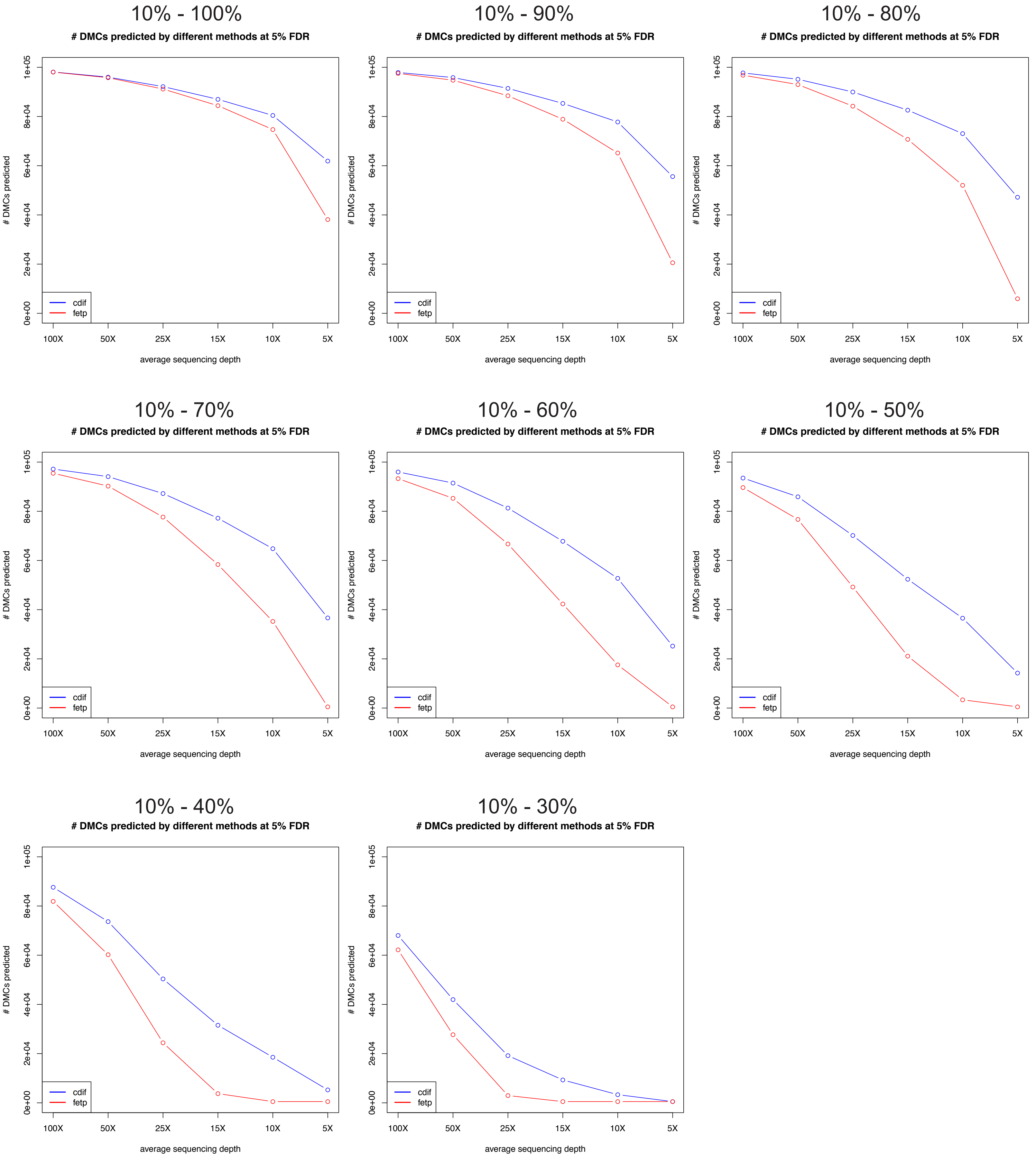

Supp Figure 2b. Sun *et al.*

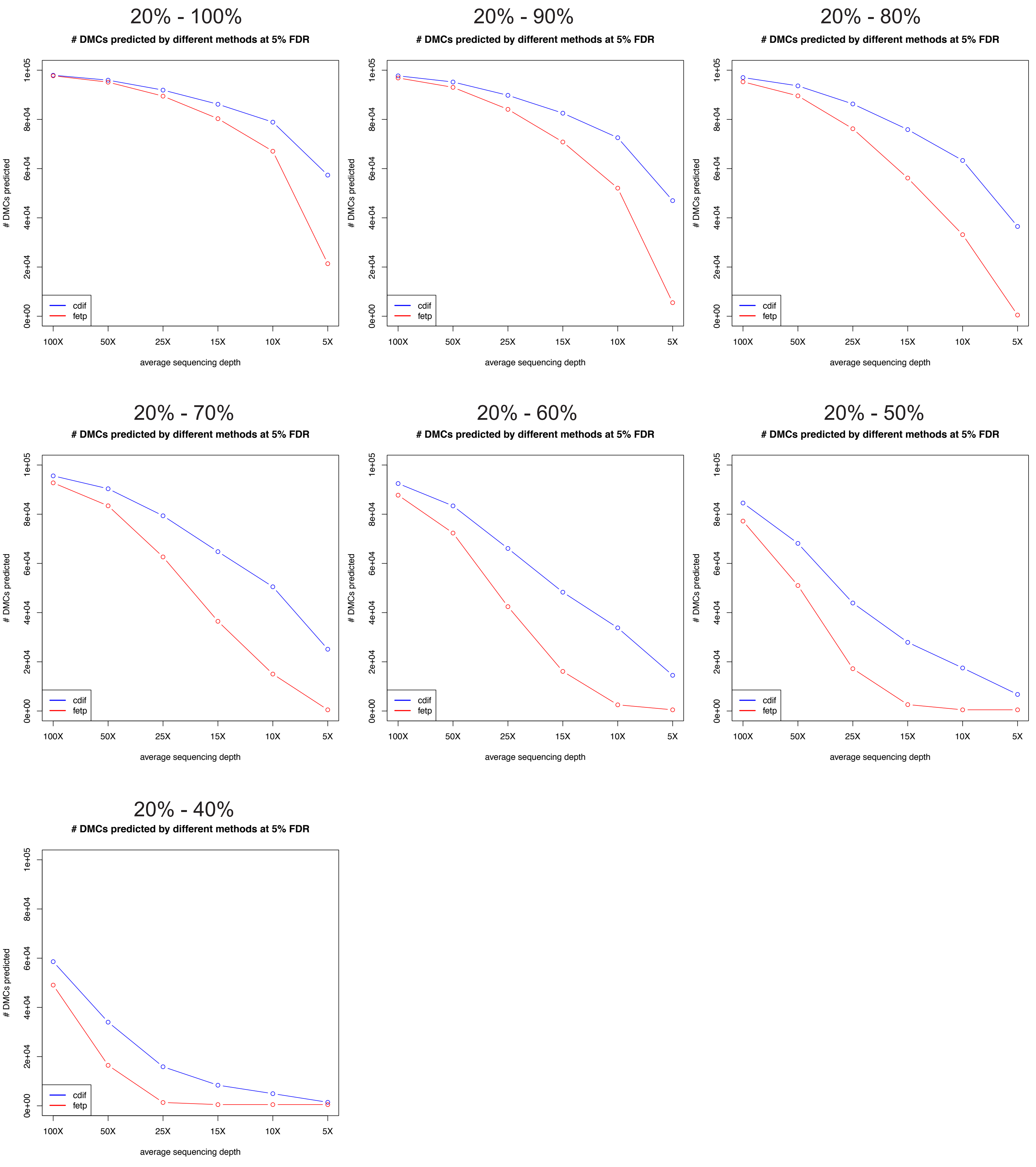

Supp Figure 2c. Sun *et al.*

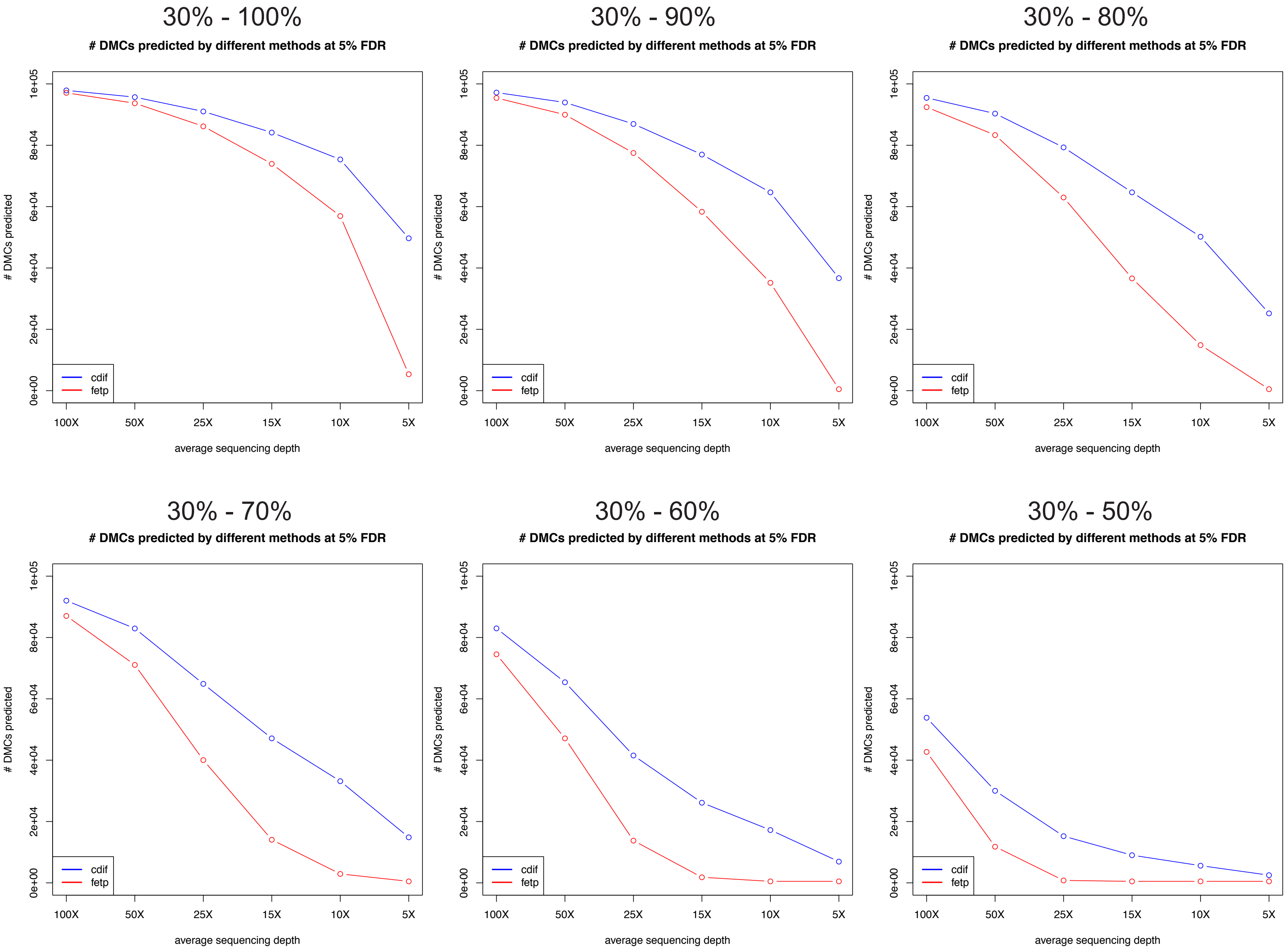

Supp Figure 2d. Sun *et al.*

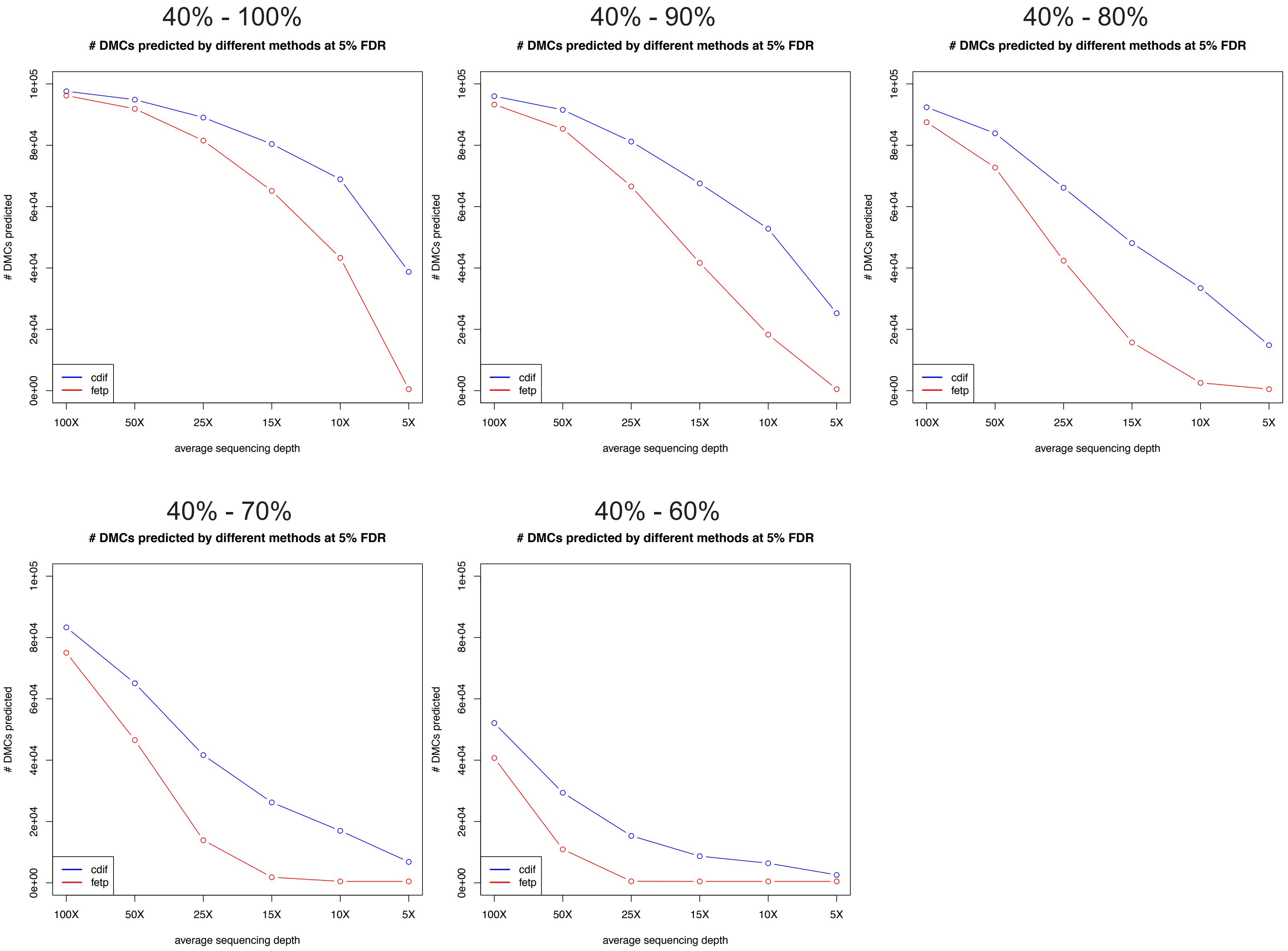

Supp Figure 2e. Sun *et al.*

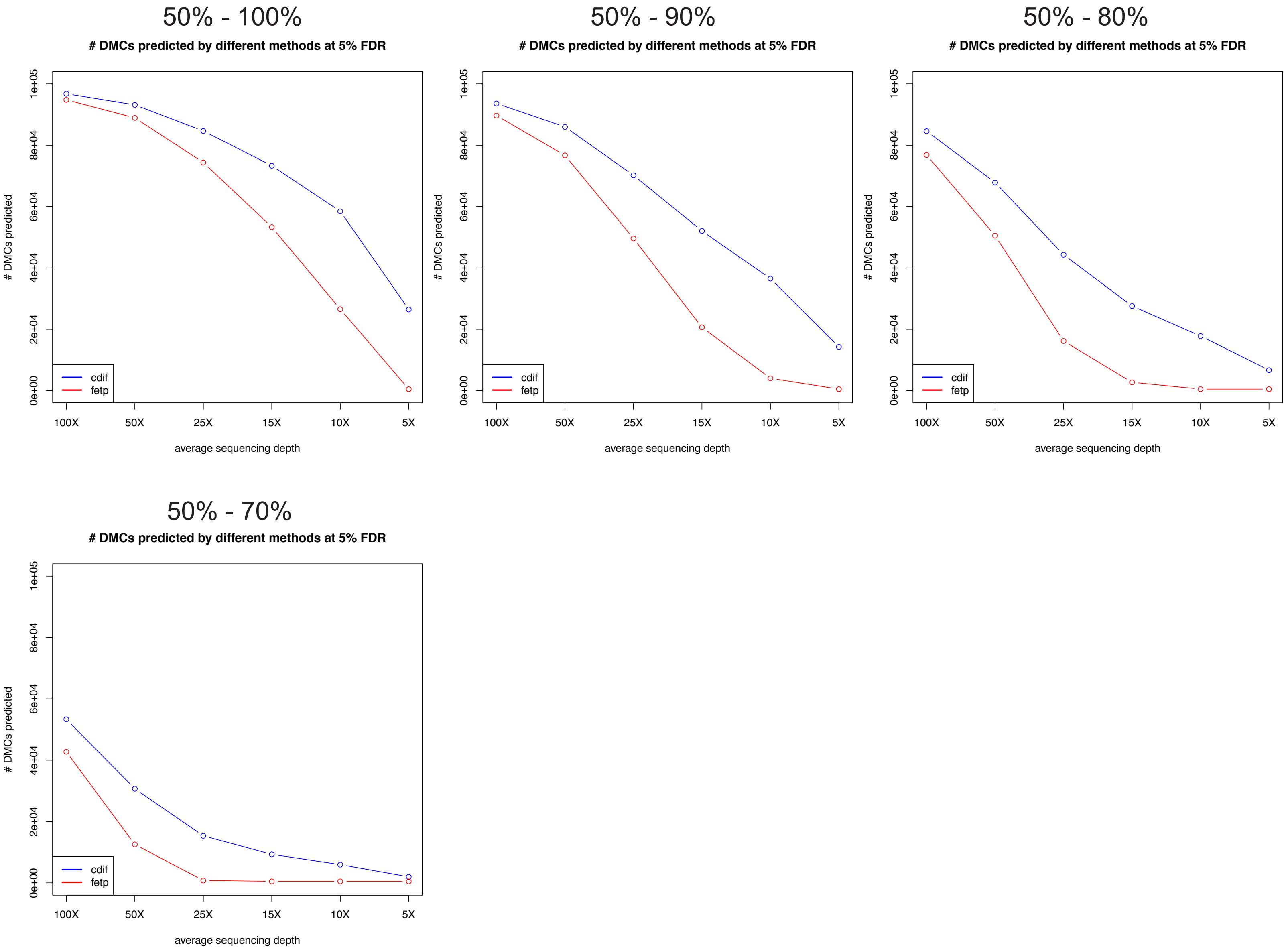

Supp Figure 2f. Sun *et al.*

DMRs predicted by Fisher's pvalue method but not reproducible between replicates

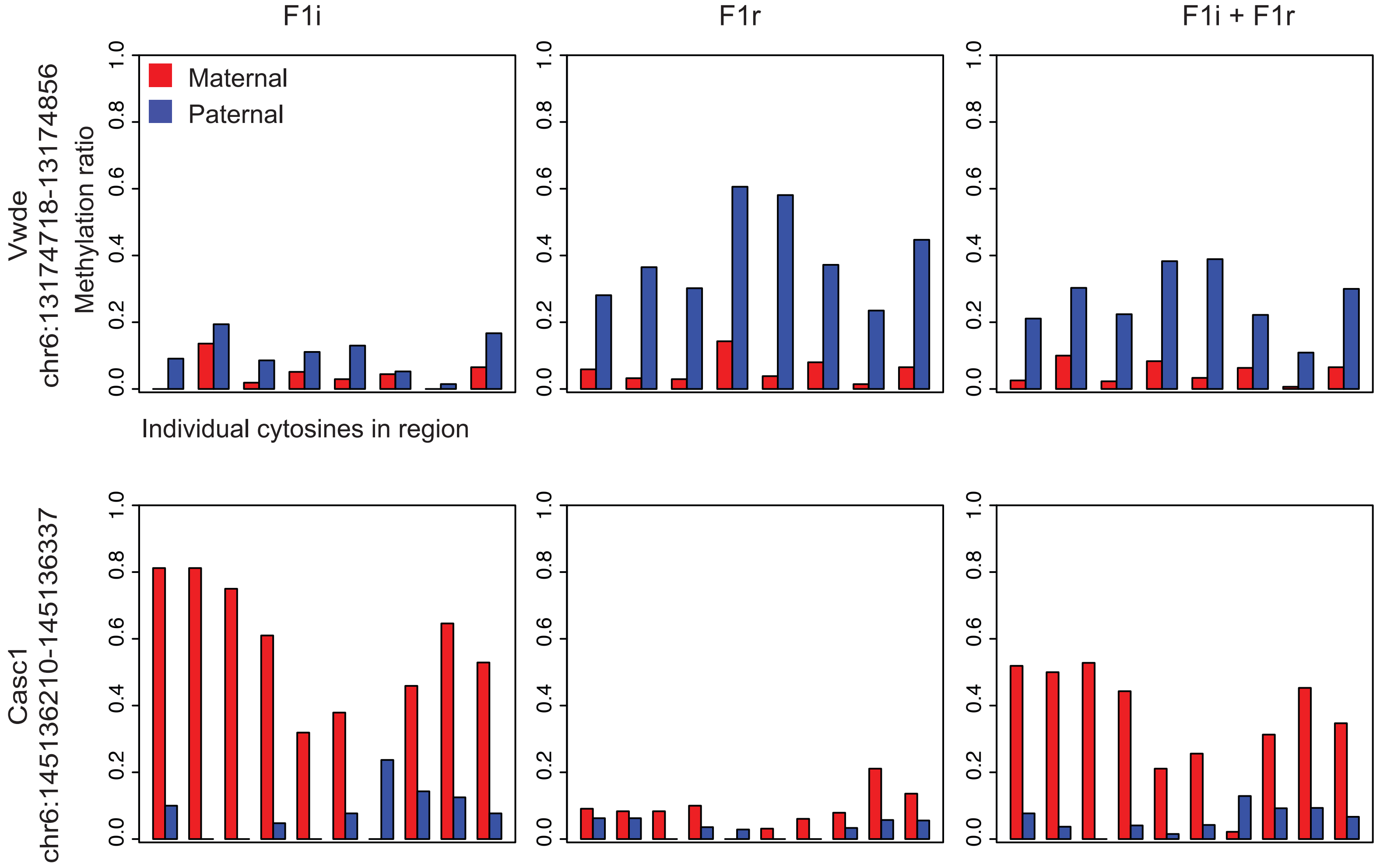

Supp Figure 3. Sun *et al.*

Novel imprinted DMRs predicted by Credible Difference method at level of Ndn and Igf2r

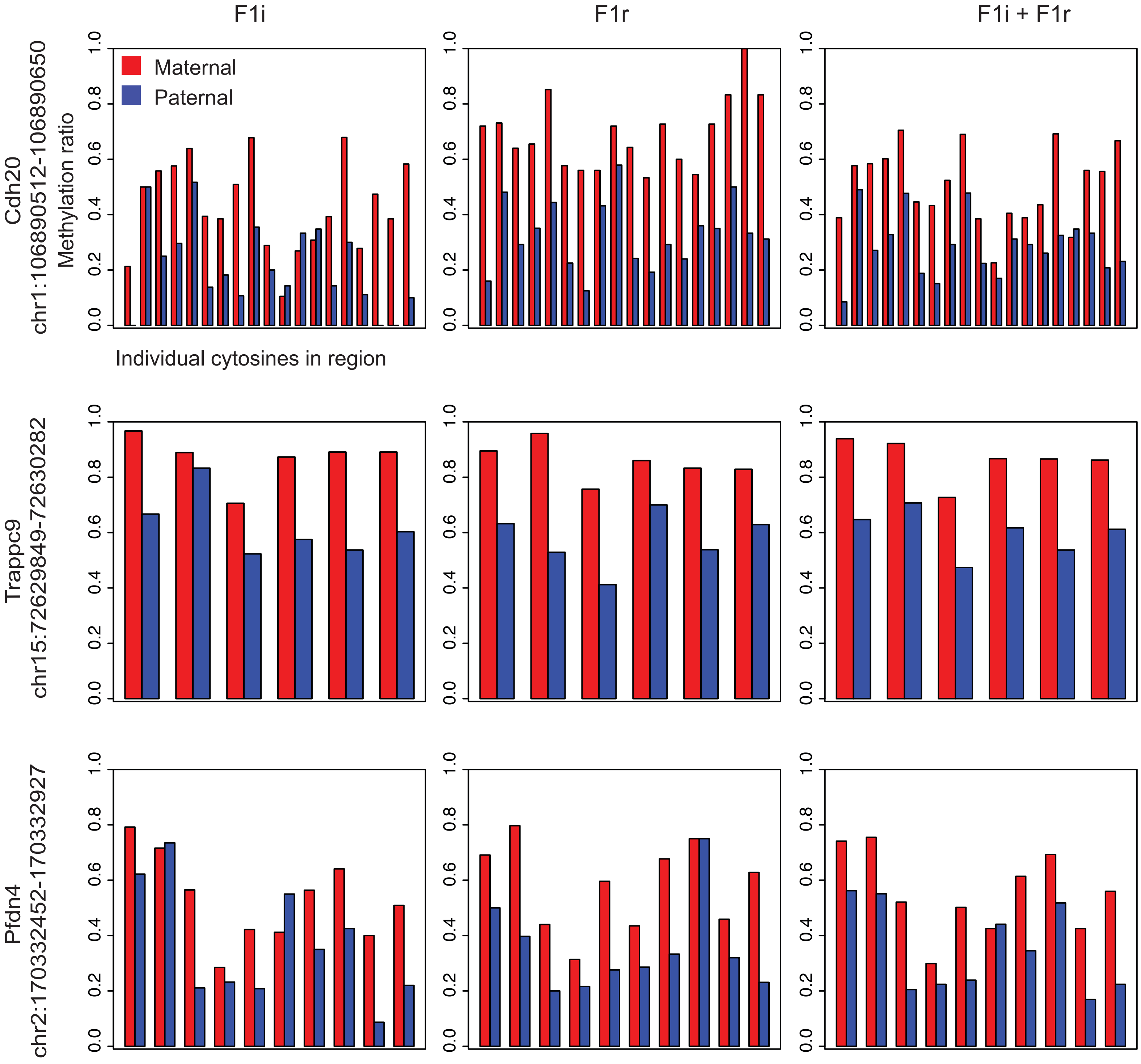

Supp Figure 4. Sun *et al.*

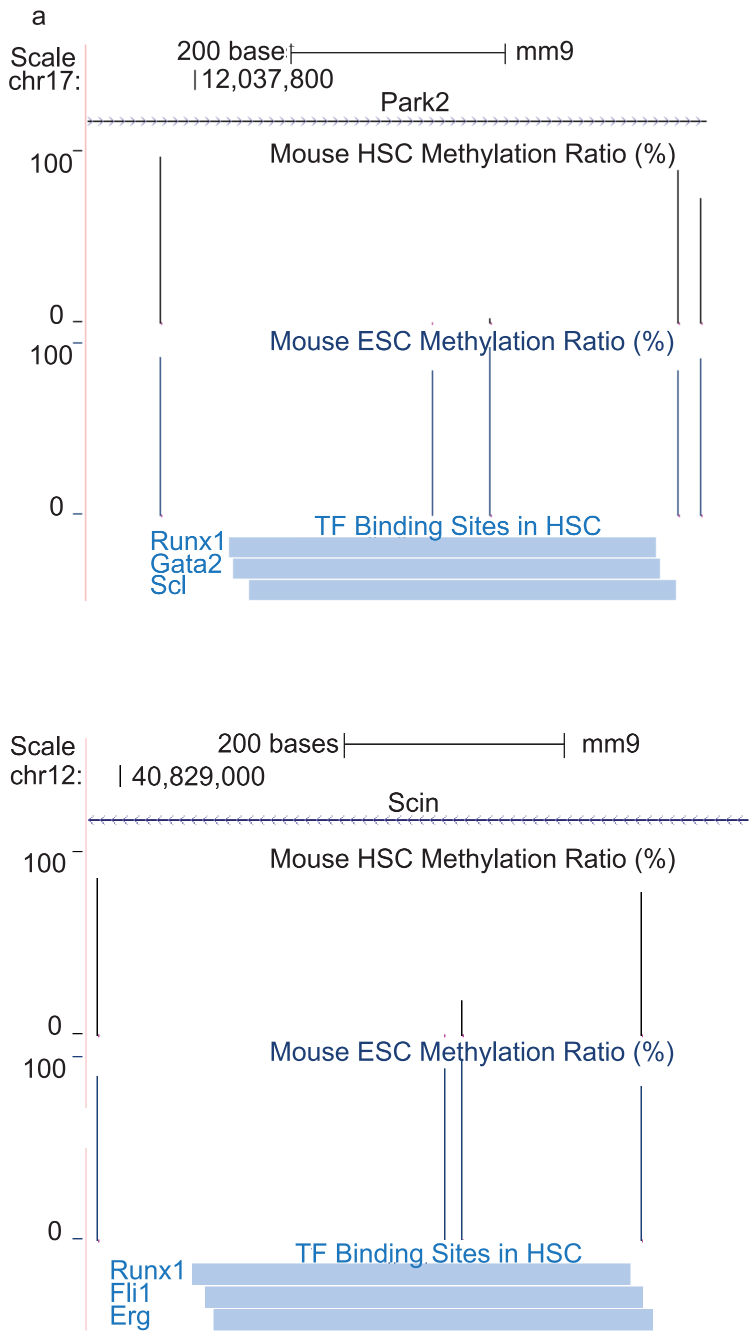

Supp Figure 5. Sun *et al.*
