# Supplementary material for: MOABS: model based analysis of bisulfite sequencing data
Source: Genome Biol. 2014 Feb 24;15(2):R38. doi: 10.1186/gb-2014-15-2-r38 (PMC4054608; doi:10.1186/gb-2014-15-2-r38)
Supplement: Additional file 4 — The testing data used for the Credible Difference method validation. [file gb-2014-15-2-r38-S4.docx]

The testing data used for the method validation is hosted at

http://dldcc-web.brc.bcm.edu/lilab/deqiangs/moabs/ simulation_validation.tar.gz

The archive contains a readme file, a result file, and multiple subfolders.
