# Supplementary material for: MOABS: model based analysis of bisulfite sequencing data
Source: Genome Biol. 2014 Feb 24;15(2):R38. doi: 10.1186/gb-2014-15-2-r38 (PMC4054608; doi:10.1186/gb-2014-15-2-r38)
Supplement: Additional file 7 — This file is the section of additional method. [file gb-2014-15-2-r38-S7.docx]

# Supplementary methods

## Methylation ratio of one locus follows a Beta Distribution

In bisulfite sequencing, one cytosine locus is sequenced $n$ times. Out of $n$ reads, $k$ reads show cytosine and $n-k$ reads show thymine as a result of bisulfite conversion of unmethylated cytosine. The methylation ratio of this locus, $p$, is inferred from the pair $(n,k)$. In other words, a population of size $s$ and true proportion $p$ is sampled $n$ times with observed success $k$. Given $s,p,n,$ the probability of obtaining $k$ successes is probability of obtaining $k$ from pool of $sp$ successes and obtaining $n-k$ failures from pool of $s(1-p)$ failures. Population size $s$ is usually the number of cells and can be considered as infinity, resulting each of $n$ trials as an independent event. So, the probability of obtaining $k$ obeys binomial distribution,

$\text{P}\left(k;p,n\right)=\begin{matrix} lim \\ s\to\text{∞} \end{matrix}\frac{C_{sp}^{k}C_{s\left(1-p\right)}^{n-k}}{C_{s}^{n}}=C_{n}^{k}p^{k}\left(1-p\right)^{n-k}$,

where $P$ is the probability, and $C_{n}^{k}$ is binomial coefficient. It is also the probability density distribution function $f(k;p,n)=C_{n}^{k}p^{i}(1-p)^{n-k}$ because it’s a discrete distribution.

This is a function of $k$ and we want to estimate the proportion $p$. Since each trial is independent and binomial, the inferred true proportion is called binomial proportion. Here, and through out this article, we estimate it regarding it as a random variable, i.e., from the Bayesian perspective. Under the uniform priori for $p$ in (0,1), the probability of$p_{0}\in(p-\frac{dp}{2},p+\frac{dp}{2})$ is

$$\begin{matrix} dP(p;n,k)=\frac{f(k;n,p)dp}{\sum_{0}^{1} f(k;n,p)dp} \\ =\frac{C_{n}^{k}p^{k}(1-p)^{n-k}dp}{\int_{0}^{1} C_{n}^{k}p^{k}(1-p)^{n-k}dp} \\ =\frac{p^{k}(1-p)^{n-k}dp}{\int_{0}^{1} p^{k}(1-p)^{n-k}dp}. \end{matrix}$$

And hence the PDF (probability density function) of $p$ is

$\text{f}\left(p;n,k\right)=\frac{d\text{P}\left(p;n,k\right)}{dp}=\frac{p^{k}\left(1-p\right)^{n-k}}{\int_{0}^{1} p^{k}\left(1-p\right)^{n-k}dp}$,

which is recognized as Beta distribution PDF

$$Be(p;\alpha,\beta)=\frac{p^{\alpha-1}(1-p)^{\beta-1}}{\int_{0}^{1} p^{\alpha-1}(1-p)^{\beta-1}dp}$$

with $\alpha=k+1,\beta=n-k+1.$

So for the methylation analysis, $k$ follows binomial distribution , and the methylation ratio for this locus, $p$ follows Beta distribution . Under a more general priori distribution like Beta distribution$p\sim Be(\alpha_{0},\beta_{0})$, distribution for $p$ is $f(p;n,k)=Be(p;\alpha,\beta),$

with $\alpha=k+\alpha_{0},\beta=n-k+\beta_{0}$.

## CI for single binomial proportion

One immediate question is what is the CI (confidence interval) of the methylation ratio. In 2008 Pires and Amado[^1^](#_ENREF_1) compared 20 methods of interval estimators for single binomial proportion. These estimators are either in analytical form with asymptotic approximations, or in numerical solutions. Since the sequencing depth could vary from one to hundreds fold, and the methylation ratio of most loci is close to either 0 or 1, the validity of asymptotic approximation become questionable. We used the exact numerical method described by Brenner and Quan^[2](#_ENREF_2)^. It is a *Bayesian* confidence interval under uniform priori. The confidence interval $(a,b)$ for proportion $p$ is straightforward when its distribution is known.

$\int_{a}^{b} f\left(p;k,n\right)dp=1-\alpha$,

where $f$ is Beta distribution PDF and $\alpha$ is type-I error, usually at 0.05.

We propose a physically meaningful “proportional area condition”, i.e., requiring the two sided tail areas being proportional to two areas under the $p$ distribution curve separated by the mean,

$\frac{P\left(p<a\right)}{P\left(p>b\right)}=\frac{P\left(p<\mu\right)}{P\left(p<\mu\right)},\text{ where }\mu=\frac{k}{n}$.

The usual choice of minimal length condition also satisfies the needs well. In the C++ and R source code two other alternative conditions are made available for general use except for DNA methylation because these two conditions, symmetric width $b-u=u-a$ and symmetric area $P(p>b)=P(p<a)$, need additional processing for the abundant situations when $p=0,\text{ or }1$.

The minimal length condition is equivalent to

$\left\{\begin{matrix} f\left(a;k,n\right)=f\left(b;k,n\right),k\neq0 \\ a=0,b=1-\alpha^{\frac{1}{n+1}},k=0 \end{matrix}\right.$.

Combining with or with , (a, b) can be uniquely solved.

The methylation ratios of millions of Cytosines in genome often obey a bimodal distribution. We may use this bimodal distribution as the priori distribution. The influence of a non-uniform priori distribution, for example a U-shape priori $p\sim Be(0.5,0.5)$, will in general make 0% methylation CI narrower but 50% methylation CI wider and have more influence at low depth than high depth.

## CI for difference of two binomial proportions in details

We showed that methylation ratio$p$with $k$methylated cytosines out of $n$ total reads, follows beta distribution from the Bayesian perspective. The probability density function is

$f(p;n,k)=Be(\alpha,\beta)=\frac{p^{\alpha-1}(1-p)^{\beta-1}}{\int_{0}^{1} p^{\alpha-1}(1-p)^{\beta-1}dp}$,

where $\alpha=k+\alpha_{0}$, $\beta=n-k+\beta_{0}$, if$Be(\alpha_{0},\beta_{0})$is priori distribution for $p$. We also give formulas to numerically calculate the confidence interval for the single binomial proportional $p$ under observed $(n,k)$.

The question is the difference of two binomial proportions, for example, the methylation ratio difference of the same genomic locus from two biological samples. Many methods have been proposed to estimate the confidence interval of $p_{1}-p_{2}$. Newcombe (1998)[^3^](#_ENREF_3) compared 11 methods, including 9 asymptotic methods and 2 exact methods, and concluded that the Wilson(1927)[^4^](#_ENREF_4) score method with modifications has superior performance. Santner *et al*. (2007)[^5^](#_ENREF_5) in a small-sample study compared the method score method with other 4 exact method and arrived at an opposite conclusion where score method is worst and the CT method Coe and Tamhane (1993)[^6^](#_ENREF_6) has best small sample performance. However Nurminen and NewCombe replied with disagreement [^7^](#_ENREF_7). Much of the debates come from different evaluation criteria, for example, whether coverage probability is minimum or average at $1-\alpha$, whether minimum CI length or symmetric tail area is looked for. Pradhan and Banerjee (2008)[^8^](#_ENREF_8) proposed a weighted likelihood method, and concluded it’s better than score method. Kawasaki[^9^](#_ENREF_9) compared several exact methods and recommended some revisions. The various methods discussed in each comparison article are just a portion of all available methods. There does not exist a comprehensive comparison of currently available methods. That motivated us to turn to the direct and exact numerical calculation of confidence interval from Bayesian perspective.

Let $t=p_{1}-p_{2}$, where $p_{i}$ is the proportion for the sample i with observation $n_{i}$ and $k_{i}$. Since the joint probability density of such observation is $f(p_{1};n_{1},k_{1})f(p_{2};n_{2},k_{2})$, the PDF for $t$ is

$$f(t)=\int_{0}^{1} dp_{2}f_{1}(p_{2}+t)f_{2}(p_{2})=\int_{0}^{1} dp_{1}f_{1}(p_{1})f_{2}(p_{1}-t),$$

where $f_{i}(p_{i})\equiv f(p_{i};n_{i},k_{i})$.

The probability

$$\begin{matrix} P(p_{1}-p_{2}>d)=P(t>d)=\int_{d}^{1} dtf(t) \\ =\int_{d}^{1} dt\int_{0}^{1} dp_{1}f_{1}(p_{1})f_{2}(p_{1}-t) \\ =\int_{d}^{1} dp_{1}f_{1}(p_{1})\int_{d}^{1} dtf_{2}(p_{1}-t) \\ =\int_{d}^{1} dp_{1}f_{1}(p_{1})\int_{p_{1}-1}^{p_{1}-d} dyf_{2}(y) \\ =\int_{d}^{1} dp_{1}f_{1}(p_{1})\int_{0}^{p_{1}-d} dyf_{2}(y) \end{matrix}$$

$$P(p_{1}-p_{2}>d)=\int_{0}^{1} dp_{1}f_{1}(p_{1})I_{2}(p_{1}-d)$$

where substitution of variable $p_{1}-t=y$ is made and$I_{2}(x)$ is cumulative distribution function for Beta distribution function$f_{2}(x)$.

Suppose the confidence interval for $t$is (a, b),

Similar conditions as in the single proportion case, like the proportional area condition, minimal length condition, can be applied to get unique solutions for (a, b).

## Identification of DMCs for two or more samples

Previously methods define a DMC by requiring methylation ratio difference, and Fisher’s exact p-value, all reach some threshold values. Now, the CDIF alone is good enough to define and rank DMCs. In MOABS, the default criteria for DMC is:

(4.1)

where $v_{0}$ is either arbitrary or determined by controlling FDR, estimated by permutation of sample labels, to be 5% (or other arbitrary cutoff). This condition may be extended to multiple samples:

$v=\max\{v_{ij}\}$ (4.2)

where $v_{ij}$denotes the credible difference between sample i and sample j.

## Identification of DMRs for two samples by simply grouping DMCs

After DMCs are identified from methylome, one may simply group DMCs into a DMR. One need specify the max gap distance between two DMCs, and how many non-differential CpGs are allowed in a DMR. The minimal number of DMCs can be determined by controlling FDR to be 5%. The NULL distribution for FDR calculation is obtained by shuffling the coordinates of all CpGs in the genome followed by DMR calling using the same method.

## Identification of DMRs for two samples by Hidden Markov Model

Here, we propose a first order Hidden Markov Model approach to combine neighboring CpGs into DMR. The state of $i^{th}$ cytosine is denoted as $S_{i}$ where $S_{i}$can take 3 hidden states for a two-sample comparison:

$S_{0}$: hypo-methylation state if $p_{2}-p_{1}<-v_{0}$;

$S_{1}$: no difference state if $\left|p_{2}-p_{1}\right|<v_{0}$; (6.1)

$S_{2}$: hyper-methylation state if $p_{2}-p_{1}>v_{0}$;

where $v_{0}$is a preset parameter and marks the characteristic threshold of difference for underlying dataset. In MOABS, this parameter is determined in DMC scan stage by controlling FDR, estimated by permutation of sample labels, to be 5%. We model the neighbor correlation by first order Markov chain

$\Pr\left(S_{i}\right)=\Pr\left(S_{i}\text{|}S_{i-1}\right)$, (6.2)

which means that the state of site i is directly influenced by previous site i-1.

Each observation for each site is a combination of 4 numbers from 2 samples: $x=(n_{1},k_{1},n_{2},k_{2})$. In this problem, we are given the observation sequence from all sites, we want to find the HMM model that maximizes the probability of observation sequence. The HMM is characterized by initial state $\pi_{0}$, transition probability matrix $A=Pr\left(S_{i}|S_{i-1}\right)$ and emission probability matrix $B=Pr\left(x_{i}|S_{i}\right)$.

The initial state $\pi_{0}$ can just takes value $S_{1}$, though its value does not matter since there are millions of CpGs in the genome. By assuming a site is in one of the three states, the emission probability for the $i^{th}$site to observe $x=(n_{1},k_{1},n_{2},k_{2})$ when the state of the site is $S_{i}$, can be derived as

$$\Pr(n_{1},k_{1},n_{2},k_{2}|s_{i})=\frac{\iint_{s_{i}} dp_{2}dp_{1}f(k_{1};n_{1},p_{1})f(k_{2};n_{2},p_{2})}{\int_{0}^{1} f(k_{1};n_{1},p_{1})dp_{1}\int_{0}^{1} f(k_{2};n_{2},p_{2})dp_{2}}$$

Since there are millions of sites and there is a high chance of repeated observations, MOABS uses a lookup table to avoid repeated computation of numerical integrations. The state transition probability matrix can be trained using the forward-backward algorithm. In the training process, the initial state, and the emission probability matrix are fixed while the state transition probability is the only model variable. Since the training is computationally intensive, MOABS may choose only a subset of all cytosine sites in the genome, like 1^st^ one million sites in chromosome 19 or locus provided by users. After the change of likelihood of the model is smaller than a given threshold or max number of iterations is reached, the optimal hidden state for each site is obtained. Consecutive sites with $S_{0}$ ( or $S_{1}$) states are merged as hypo-DMR ( or hyper-DMR).

## Identification hypo-methylated regions from one sample

Similar to DMR detection, MOABS used a two-state first order Hidden Markov Model (HMM) to detect highly methylated and lowly methylated regions from a single sample. Random shuffle of all the CpGs in the genome, followed by the same procedure, generates a NULL distribution to control the FDR.

Reference

1. A.M. Pires; C. Amado Interval estimators for a binomial proportion: comparasion of twenty methods. *REVSTAT* **6** (2008).

2. Quan, D.J.B.H. Exact confidence limits for binomial proportions—Pearson and Hartley revisited. **39**, 391-397 (1990).

3. RG, N. Interval estimation for the difference between independent proportions: comparison of eleven methods. *Stat Med* **17** (1998).

4. Wilson, E.B. Probable inference, the law of succession, and statistical inference. *Journal of the American Statistical Association* **22** (1927).

5. Santner, T.J., Pradhan, V., Senchaudhuri, P., Mehta, C.R. & Tamhane, A. Small-sample comparisons of confidence intervals for the difference of two independent binomial proportions. *Computational Statistics & Data Analysis* **51**, 5791-5799 (2007).

6. Tamhane, P.R.C.A.C. Small sample confidence intervals for the difference,ratio and odds ratio of two success probabilities. *Communications in Statistics - Simulation and Computation* **22** (1993).

7. Newcombe, M.M.N.R.G. Score intervals for the difference of two binomial proportions. *METHODOLOGIC NOTES ON SCORE INTERVALS*.

8. Pradhan, V.B., Tathagata Confidence interval of the difference of two independent binomial proportions using weighted profile likelihood. *COMMUNICATIONS IN STATISTICS-SIMULATION AND COMPUTATION* **37**, 645-659 (2008).

9. Kawasaki, Y. COMPARISON OF EXACT CONFIDENCE INTERVALS FOR THE DIFFERENCE BETWEEN TWO INDEPENDENT BINOMIAL PROPORTIONS. *Advances and Applications in Statistics* **15**, 157-170 (2010).
